# Probing conformational changes during activation of ASIC1a by an optical tweezer and by methanethiosulfonate-based cross-linkers

**DOI:** 10.1371/journal.pone.0270762

**Published:** 2022-07-08

**Authors:** Anand Vaithia, Stephan Kellenberger

**Affiliations:** Department of Biomedical Sciences, University of Lausanne, Lausanne, Switzerland; University of Missouri-Kansas City, UNITED STATES

## Abstract

Acid-sensing ion channels (ASICs) are neuronal, proton-gated, Na^+^-selective ion channels. They are involved in various physiological and pathological processes such as neurodegeneration after stroke, pain sensation, fear behavior and learning. To obtain information on the activation mechanism of ASIC1a, we attempted in this study to impose distance constraints between paired residues in different channel domains by using cross-linkers reacting with engineered Cys residues, and we measured how this affected channel function. First, the optical tweezer 4′-Bis(maleimido)azobenzene (BMA) was used, whose conformation changes depending on the wavelength of applied light. After exposure of channel mutants to BMA, an activation of the channel by light was only observed with a mutant containing a Cys mutation in the extracellular pore entry, I428C. Western blot analysis indicated that BMA did not cross-link Cys428 residues. Extracellular application of methanethiosulfonate (MTS) cross-linkers of different lengths changed the properties of several Cys mutants, in many cases likely without cross-linking two Cys residues. Our observations suggest that intersubunit cross-linking occurred in the wrist mutant A425C and intrasubunit cross-linking in the acidic pocket mutant D237C/I312C. In these mutants, exposure to cross-linkers favored a non-conducting channel conformation and induced an acidic shift of the pH dependence and a decrease of the maximal current amplitude. Overall, the cross-linking approaches appeared to be inefficient, possibly due to the geometrical requirements for successful reactions of the two ends of the cross-linking compound.

## Introduction

**Fig 1:**
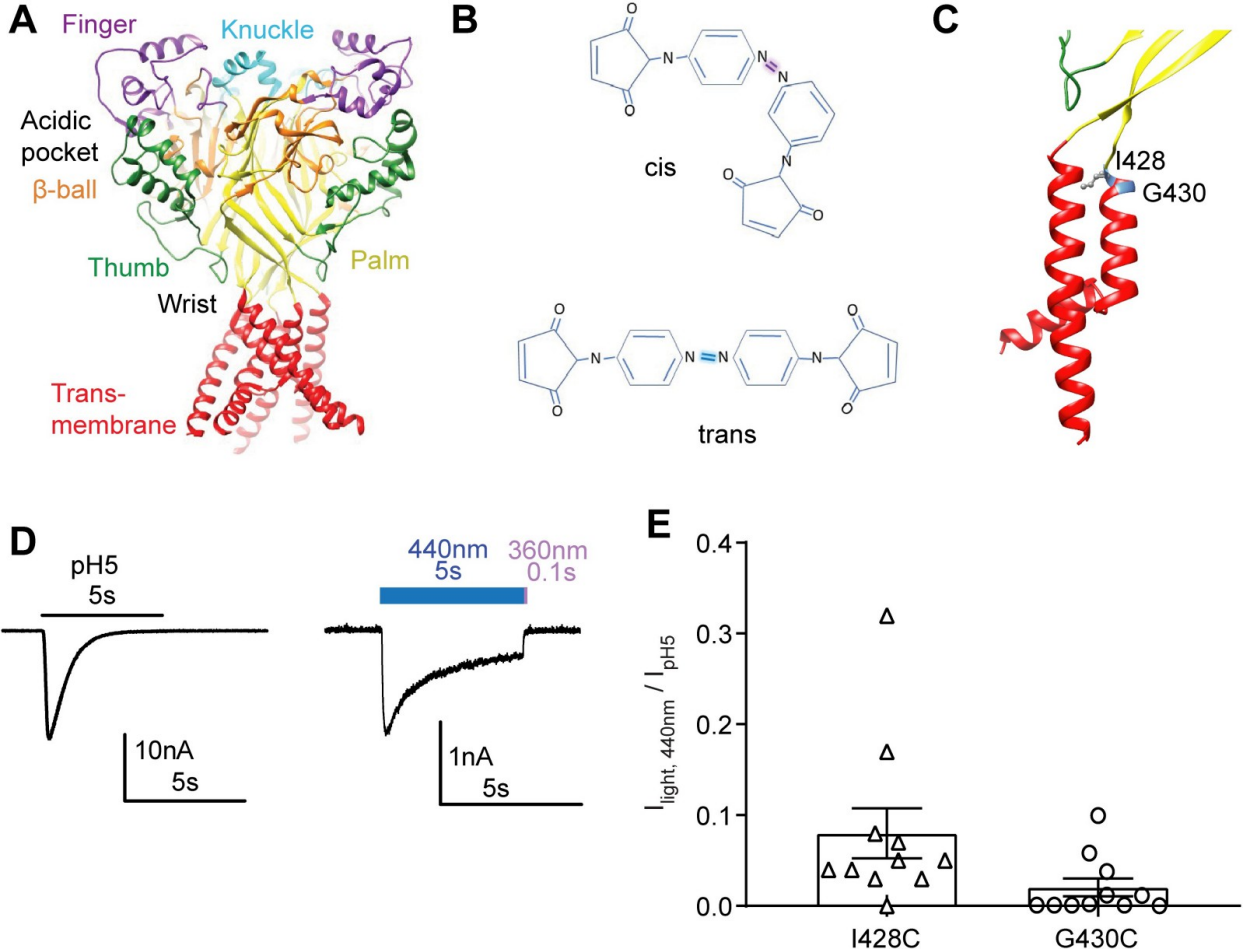
Light-dependent activation of ASIC1a. **A**, Structural image of the ASIC1a trimer showing its domain organization; transmembrane (red), thumb (green), palm (yellow), β-ball (orange), finger (purple), and knuckle (cyan; human ASIC1a model based on chicken ASIC1a structure). **B,** BMA in *cis* (top) and *trans* conformation (bottom). **C**, Localization of I428 and G430 in the transmembrane 2 domain. **D-E**, data obtained from whole-cell patch-clamp recording of transfected CHO cells, voltage-clamped to -60mV. **D**, Representative traces of pH5-induced (left) and 440nm light-induced current (right) of hASIC1a-I428C. The blue and purple bars over the right trace represent the application of 440nm light (5s) and 360nm light (0.1s), respectively. **E**, Light-induced current amplitudes measured from the indicated mutants, normalized in each cell to the pH5-induced current amplitude; n = 11.

Acid-sensing ion channels (ASICs) are proton-gated voltage-independent Na^+^-selective ion channels that form a sub-family within the Epithelial Na^+^ Channel/Degenerin family. In rodents, 6 homologous ASIC subunits, ASIC1a, -1b, -2a, -2b, -3 and -4 have been identified. Homotrimeric or heterotrimeric assembly of ASIC subunits results in channels with different pH sensitivity and kinetics [1, 2]. ASIC1a, -2a, -2b and -4 are expressed in the CNS, and all ASICs except ASIC4 are expressed in the PNS [2, 3]. ASICs are involved in various pathological and physiological functions, such as learning and memory [4–7], anxiety and fear [3], neurodegeneration after ischemic stroke [8], seizure termination [9] and pain sensation [10, 11]. Extracellular acidification results in rapid ASIC activation, producing an inward current that is transient because it decays when the channel enters the non-conducting desensitized state [2, 3, 12]. High-resolution structures of chicken ASIC1a in closed, toxin-opened and desensitized conformations [13–18] and of human ASIC1a in the closed state [19] have been published and indicate that ASICs are trimers. The structure of a single ASIC subunit has been compared to the shape of a hand holding a small ball (Fig 1A), with the two transmembrane domains corresponding to the forearm [16]. The region just above the pore entry is called "wrist". The extracellular sub-domains were named accordingly as palm, finger, knuckle, thumb and β-ball. In each subunit, the finger, thumb and β-ball enclose together with the palm of a neighboring subunit a vestibule containing several acidic amino acid residues, the acidic pocket. Structural comparison of the closed and open state indicates that during channel activation the acidic pockets collapse, the extracellular fenestrations (located just above the membrane) are expanded, and the channel gate opens [18]. During desensitization, the lower palm domains collapse towards the central vertical axis and the transmembrane helices relax back to a resting-like conformation in which the pore is closed [14, 15, 18, 20]. The acid-induced collapse of the acidic pocket was initially proposed as the driving force for channel opening [16]. The observation that simultaneous mutation of all acidic amino acid residues of the acidic pocket resulted in channels that were still activated by acidification, suggested however that protonation of acidic pocket residues is not required for ASIC activation [21]. Several mutagenesis studies identified residues outside the acidic pocket, whose mutation changed the activation pH dependence. The identification of such residues [22–28] highlighted the potential roles of the wrist and palm as pH-sensing domains.

The present study combines functional analysis with the application of Cys-reactive cross-linkers to mutant ASIC1a channels containing engineered Cys residues in the wrist and extracellular domains, in order to identify conformational changes that are involved in ASIC1a activation. Experiments were carried out with a cross-linker whose conformation can be changed by light, and by a series of light-insensitive cross-linkers of different length. Channels were exposed to these cross-linkers with the aim of locking the channel in specific functional states. Cross-linking appeared to be inefficient; for many mutants and reagents however, it appeared that reaction of a single end of these compounds with a Cys residue affected ASIC function and identified residues involved in activation and desensitization.

## Materials and methods

### Reagents

4, 4′-Bis (maleimido) azobenzene (BMA) and MTS (methanethiosulfonate) compounds were purchased from Toronto Research Chemicals (Canada). BMA was dissolved in dimethylsulfoxide (DMSO) to prepare a 10mM stock solution, which was stored in the dark at -20°C. Stock solutions of MTS compounds at 100mM were prepared for MTS-14-PEO3-MTS and MTS-17-PEO5-MTS in methanol, for MTS-11-MTS in chloroform and for all other MTS reagents in DMSO and stored at -20°C. The stock solutions were thawed at the beginning of the electrophysiological experiments and kept at 4°C.

### Molecular biology

Cysteine mutations were introduced in human ASIC1a [29, 30] and human ASIC2a [29] in the mammalian expression vector peak8 (Edge Biosystems, Gaithersburg, MD), using site-directed mutagenesis. The human ASIC1a clone used contained a Gly residue at position 212 [30]. All except two mutants were generated in this background. Two single mutants used here, D237C and D351C, had previously been generated in the D212 background [29, 30]. Primers for the mutagenesis were designed using the Quikchange site-directed mutagenesis method in the PrimerX online tool and synthesized by Microsynth (Switzerland). Site-directed mutagenesis was performed using KAPA HiFi Hot-start PCR polymerase (Roche, Switzerland). All mutations were confirmed by Sanger sequencing (Synergene and Microsynth, Switzerland). The rat P2X2 P329C mutant in pcDNA3.1 was a gift of Dr. Thomas Grutter (University of Strasbourg, France).

ASIC1a Cys mutants in the peak8 vector were sub-cloned in the pSP65-derived vector pSD5, containing 5’ and 3’ non-translated sequences of β-globin for improved protein expression in *Xenopus laevis* oocytes. The sub-cloning was done with the cloning kit In-Fusion HD (TaKaRa, Switzerland), using digestion of the pSD5 vector by EcoRI and XbaI, and was verified by analytical digestions and sequencing. cRNA was synthesized using the *in vitro* transcription kit mMESSAGE mMACHINE™ SP6 (ThermoFisher, Switzerland). Integrity of the cRNA was verified by running the synthesized cRNA on an agarose gel.

### Mammalian cell culture and transfection

Wild-type and mutant ASIC1a or ASIC2a were transiently co-transfected with EGFP or sfGFP in Chinese hamster ovary (CHO) cells using Rotifect (CarlRoth, Karlsruhe, Germany), following the standard manufacturer protocol. Since recent studies had shown successful application of optical tweezers to P2X2 receptors [31, 32], the mutant P329C of rat P2X2 was used as a positive control to validate the measuring setup. For the expression of rP2X2-P329C, HEK cells were transiently co-transfected with EGFP. The ratio of EGFP to ASIC or P2X2 was 0.2:1. CHO cells were cultured in DMEM/Nutrient Mixture F-12, HEK cells in DMEM medium; these media were supplemented with GlutaMax^TM^ medium, 10% fetal bovine serum (FBS, ThermoFischer Scientific) and 1% Penicillin/Streptomycin (5,000 U/mL, ThermoFischer Scientific). Cells were grown at 37°C in a 5% CO_2_ atmosphere, and currents were measured 48–72 h after transfection.

### Handling and labeling of Xenopus laevis oocytes

Female South African Clawed frogs *Xenopus laevis* were anaesthetized with 1.3g/L of MS-222 (Sigma-Aldrich, USA). A small incision of ~1cm was performed on the abdominal wall for the extraction of oocytes. All procedures with Xenopus *laevis* were approved by the local veterinary authority of the Canton de Vaud, Switzerland. Healthy stage V and VI oocytes were treated with collagenase for isolation and defolliculation. Oocytes were injected with 50nl cRNA at concentrations of 10–500 ng/μl. Oocytes were stored during protein expression in modified Barth’s saline (MBS) containing (in mM) 85 NaCl, 1 KCl, 2.4 NaHCO_3_,0.33 Ca (NO_3_)_2_,0.82 MgSO_4_,0.41 CaCl_2_, 10 HEPES, and 4.08 NaOH.

### Electrophysiology

#### Patch-clamp

Whole-cell patch-clamp recordings were carried out at -60mV at RT. The BMA stock solution was diluted to different concentrations (10–40 μM) in the extracellular solution at pH7.3 immediately before use, and the cells were labelled at room temperature in the dark for 12, 20 or 40min. Unless noted differently, the BMA concentration used was 20mM, and the exposure time was 20min. The cells were subsequently washed twice with the extracellular solution at pH 7.4 and were used immediately in the experiment. Whole-cell recordings were performed using an EPC-10 patch-clamp amplifier and the PatchMaster software (HEKA Electronics, Germany). The solution exchange for the experiments was done using a cFlow 8 channel flow controller connected to the MPRE8 perfusion head (Cell MicroControls, Virginia, USA). The sampling interval for all the experiments was set at 1ms and low-pass filtering at 3 kHz. Patch pipettes had a resistance between 3–4MΩ when filled with intracellular solution. The pipette solution contained (in mM): 90 K-gluconate, 10 NaCl, 10 KCl, 60 HEPES, 10 EGTA, with a final osmolarity of 290 mOsm, adjusted to pH7.3 with KOH. The standard extracellular solution contained (in mM): 140 NaCl, 4 KCl, 2 CaCl_2_, 1 MgCl_2_, 10 MES, 10 HEPES, 10 Glucose to a final osmolarity of 320 mOsm, adjusted to the desired pH with NaOH or HCl. For NMDG^+^-containing extracellular solution, NaCl was replaced by NMDG^+^. Illumination of cells was achieved with high-power LEDs of 445nm and 365 nm wavelengths (SOLIS, ThorLabs). Light was directed on the cells using a CFI S Fluor 40X Plan Fluorite 40X, 0.75 NA objective lens (Nikon). The intensity of the light output was measured using a handheld optical meter (Newport). The maximal measured output intensities for wavelengths of 360 and 440 nm were 2.8 and 6.3 mW/mm^2^ (corresponding to the maximal intensity). Unless noted differently, the 440nm light was applied at 10% of the maximal intensity, while the 360nm light was applied at 100% of the maximal intensity. To validate the measuring setup, the previously reported mutant P2X2-P329C [31, 32] was tested. In this mutant, the peak current amplitude induced with 440nm light was ~15% of that induced with 7mM ATP (S1 Fig).

#### Recordings using *Xenopus* oocytes

Electrophysiology experiments were performed 1–2 days after cRNA injection with a Dagan TEV-200 amplifier (Minneapolis, MN) equipped with two bath electrodes at a holding potential of -60mV. Oocytes were placed in the recording chamber, impaled with two glass electrodes filled with 1M KCl with a resistance of <0.5 MΩ and perfused with experimental solutions by gravity at a rate of 10-12ml/min. The recording solution contained (in mM): 110 NaCl, 2 CaCl_2_, 10 HEPES for pH ≥ pH6.8 (MES for pH<6.8). The pH was adjusted using NaOH or HCl. Solution flow was controlled by a cFlow 8 channel flow controller and electro valve unit. Currents were recorded with the Clampex 9.2 software (Molecular Devices). The sampling interval for all the experiments was set at 1 ms and current filtering at 3 kHz. The conditioning pH in these experiments was pH7.4. It was applied during 50s between 10-s applications of acidic solutions. The MTS stock solutions were diluted in extracellular solution at pH7.4 immediately before use. Oocytes were incubated for 3min with 1mM of monovalent or cross-linking MTS reagent in the measuring chamber. Following MTS reagent treatment, oocytes were washed for 1min with extracellular solution pH7.4 before current recording.

### Cell surface cross-linking, biotinylation and Western blot

CHO cells were transiently transfected with 10μg of WT or mutant ASIC1a per 10cm cell culture plate. 48h after transfection, the cells were washed twice with extracellular solution pH7.3. The cells were then labelled in the dark with 20μM of BMA for 20 min as described above. The following steps were all done on ice. After the labelling, cells were washed with extracellular solution at pH7.3, followed by two washing steps with ice-cold PBS-CM (in mM, 137 NaCl, 2.7 KCl, 8 Na_2_HPO_4_, 2 KH_2_PO_4_, 0.1 CaCl_2_, 1 MgCl_2_) at pH7.4, followed by two wash steps with PBS-CM at pH8.0. Cells were incubated with EZ-link Sulfo-NHS-SS-Biotin (ThermoFischer Scientific) in PBS-CM, pH8, at a concentration of 1mg/ml for 15 min. Biotin was quenched by PBS-CM containing 100mM glycine for 10 min. The cells were then washed twice with PBS-CM at pH7.4. The cell lysate was prepared by scraping the cells in 1 ml of membrane isolation buffer containing (in mM) 100 NaCl, 5 EDTA, 20 HEPES, 1% Triton X-100 at pH 7.4, supplemented with 200mM cOmplete protease inhibitor (Roche). Lysed cells were centrifuged at 11’000g, 4°C, and the supernatant was collected. The supernatant was added to 50 μl of Streptavidin Agarose resin beads (ThermoFischer Scientific) and samples were incubated overnight at 4°C on a rotating wheel. On the next day, beads were washed thrice with ice-cold PBS-CM at pH7.4 and recovered by centrifugation at 4°C, 1000g. Recovered beads were added to 50 μl of 2x sample loading buffer (20% glycerol, 6% SDS, 250mM Tris-HCl at pH6.7, 0.1% (w/v) bromophenol blue, 50mM DTT) and surface protein was kept at 65°C for 15min. Surface protein samples of 25μl were loaded and resolved on Mini-protean TGX stain free 4–15% precast SDS-PAGE (BioRad) in running buffer containing 27.5mM Tris-base, 213mM Glycine and 1% SDS at 100V for 1.5h. Proteins were transferred to ProtranTM 0.2μM nitrocellulose membranes (Amersham Biosciences) at 4°C and 100V for 2.5h. After the transfer, the membrane was blocked by TBST (137mM NaCl, 2.7mM KCl, 19mM Tris-base, 0.1% Tween 20) containing 5% non-fat milk for 1h. Membranes were exposed overnight at 4°C to polyclonal anti-ASIC1 antibody MTY19 (1:1000) [33] in TBST buffer containing 1% non-fat milk, washed three times, and were then exposed to Donkey anti-rabbit IgG horseradish peroxidase-linked secondary antibody (1:4000, GE healthcare, Switzerland). The antibody MTY19 is directed to the C-terminal 22 amino acid residues of mouse ASIC1a; it has been shown to detect ASIC1a with high specificity in the hippocampus [33]. To detect actin, the same blots were exposed overnight at 4°C to Anti-actin (1:1000, Sigma Aldrich) in TBST buffer containing 1% BSA, washed three times and detected with Donkey anti-rabbit IgG horseradish peroxidase linked secondary antibody (1:4000, GE healthcare). Blots were exposed to secondary antibodies for 1h at room temperature and washed three times with 1x TBST. The signals were detected using the Fusion SOLO chemiluminescence system (Vilber Lourmat, Marne-laVallée, France) using SuperSignalTM West Femto maximum sensitivity substrate (Thermo Scientific). The band intensities were quantified by the linear analysis method of the software, with the area of measurement kept constant for all samples of the same blot. Background noise was subtracted prior to determining the intensity of individual bands.

### Structural models

The crystal structures of chicken ASIC1a in open, desensitized and closed state (PDB ID: 4NTW, 4NYK and 5WKU; [14, 15, 18]) were used to generate homology models of human ASIC1a, as described [34]. Distances in the models were measured by using Chimera [35].

### Data analysis

The pH of half maximal activation (pH_50_) was determined by fitting normalized activation curves to the Hill equation, I = I_max_/(1+(10^-pH50^/10^-pH^)^nH^), where I_max_ is the maximal current amplitude, pH_50_ is the pH that induces a current amplitude corresponding to 50% of the maximal current amplitude, and *n*H is the Hill-coefficient. Data are presented as mean ± SEM. Differences between ASIC1a WT and mutant, and between different treatments were analyzed by ANOVA followed by the indicated post hoc test, using GraphPad Prism. Differences in paired experiments were analyzed with student’s t-test.

## Results

### Light-activated current in ASIC1a-I428 after incubation with BMA

A recent study showed that exposure of the mutants I428C and G430C of human ASIC1a to the optical tweezer BMA allowed the activation of the channel by light [31]. Here, we used this approach at different positions of ASIC1a to probe for conformational changes involved in ASIC activation. The end-to-end length of BMA is ~13Å in the *cis*, and ~22 Å in the *trans* conformation [31], illustrated in Fig 1B. The two mutants ASIC1a-I428C and -G430C were used as positive controls in a first set of experiments with BMA. Structural models of human ASIC1a (*Methods*) indicate an intersubunit distance between Ile428 residues (measured between the b-carbon atoms) of 21.3Å in the closed and 28.4Å in the open state; for Gly430 the corresponding distances (between a-carbon atoms) are 9.5Å and 16.6Å, respectively (Table 1). These residues are located at the top of the TM2, in the extracellular pore entry, with Ile428 pointing in the open conformation towards the outside, and Gly430 towards the adjacent subunit (Fig 1C). The I428C and G430C mutants were expressed in CHO cells, and cells were incubated with 10μM BMA for 12 min in the dark immediately before testing in whole-cell patch-clamp for two possible ways of activation, extracellular acidification, or illumination with light of 440nm wavelength. Light with a wavelength of 440nm induces the *trans* conformation of BMA [31]. Changing the extracellular pH from 7.4 to 5.0 induced transient inward currents, as shown for I428C in Fig 1D (left trace). The pH of half-maximal activation (pH_50_) of I428C, determined from exposure to solutions of different pH, was 6.46±0.03 (n = 7; S2A Fig). Application of 440nm light for 5s to I428C-expressing cells induced inward currents containing a transient and a sustained component (Fig 1D, right). Switching from 440nm to 360nm light for 0.1s brought the current back to the baseline, due to induction of the *cis* conformation of BMA and likely subsequent closure of the channel. The light-induced currents had smaller amplitudes than the pH5-induced I428C currents (Fig 1E, left). Channel activation occurred rapidly with both ways of activation, with time constants of τ = 48±16ms (440nm light) and τ = 106±24ms (pH5, n = 8). In control experiments with cells expressing ASIC1a wild type (WT) that were exposed during 12min to 10mM BMA, 440 nm light did not induce any current (n = 7), while the pH5-induced current amplitude was -4.2±1.1nA (n = 7). In contrast to a previous study [31], we were not able to induce current with 440nm light in the G430C mutant. The pH5-induced current amplitude in G430C-transfected cells was -18.1±6.9nA (n = 11), while the 440nm light-induced current amplitude was with -0.028±0.008 (n = 11) only 0.15% of the pH5-induced current amplitude (Fig 1E, right).

**Table 1:** Distance in ASIC1a WT between mutated residues.

Residue	Open 4NTW (Å)	Des. 4NYK (Å)	Closed 5WKU (Å)	Residues	Open 4NTW (Å)	Des. 4NYK (Å)	Closed 5WKU (Å)
E63	27.0	22.2	22.2	E97/D347	12.5	12.8	14.8
R64	21.0	15.0	15.2	E97/V354	17.2	17.2	21.2
Y67	24.8	18.0	17.7	E97/E355	19.8	19.7	25.4
H72	22.2	15.5	15.2	I137/K396	20.7	21.0	21.0
T419	17.3	17.9	18.8	I137/E403	20.4	20.3	21.2
E421	17.6	16.3	15.9	E235/E355	15.3	15.4	25.9
K423	16.3	12.1	11.8	T236/D351	11.4	11.4	17.4
A425	16.2	9.2	9.0	D237/F257	15.5	16.3	22.3
E427	23.8	15.6	16.0	D237/I312	17.0	17.0	23.4
I428	28.4	21.0	21.3	D237/E315	13.7	14.1	20.6
G430	16.6	9.4	9.5	D237/E355	11.6	11.6	18.2
G433	14.4	5.2	5.7	K246/D347	17.6	17.7	20.7
				F257/D351	14.6	14.3	18.0
				D296/E359	14.4	22.8	23.6

Distances between residues that were mutated to Cys in this study, determined between Cβ-atoms (Ca for Gly) of open (4NTW, [14]), desensitized (4NYK, [14, 15]) and closed state (5WKU, [18]) models of human ASIC1a. The indicated distances were measured in hASIC1a models between two subunits for single mutants and within a single subunit for double mutants.

Mutations homologous to hASIC1a-I428C and -G430C were also generated in human ASIC2a (V425C and A427C) and it was tested whether 440 nm light induced currents after exposure to BMA. This was however not the case (S3 Fig).

### Characterization of the light-activated ASIC1a-I428C current

**Fig 2:**
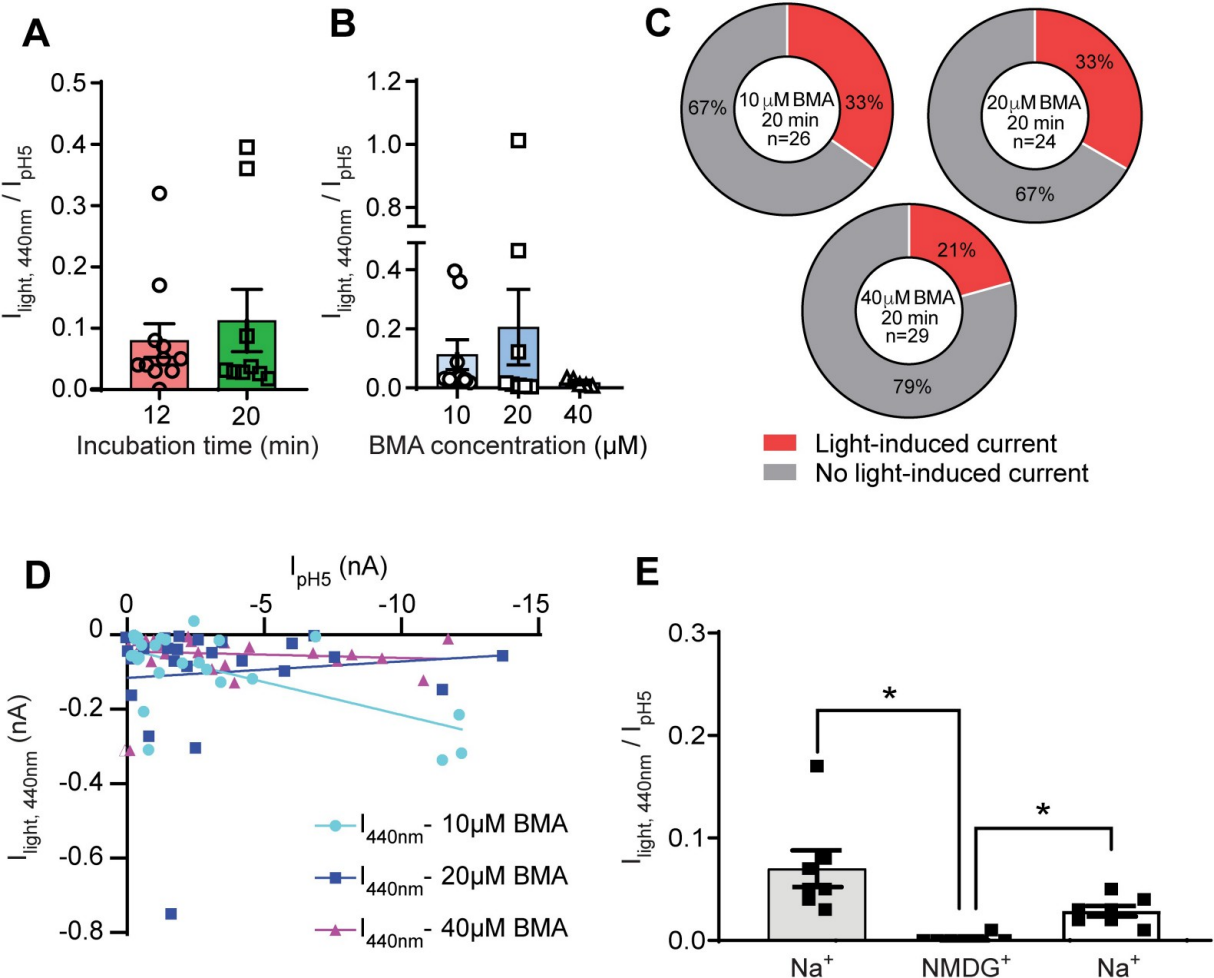
Characterization of light-activated currents in I428C. Data were obtained from whole-cell patch-clamp recording of hASIC1a-I428C-expressing CHO cells, voltage-clamped to -60mV. **A**, Light-induced current amplitudes normalized to the IpH5 measured in the same cells, obtained after incubation with 10μM BMA for 12 or 20 min. **B,** Light-induced current amplitude normalized to IpH5 measured in the same cell, obtained after incubation of I428C with different concentrations of BMA for 20min. **C**, Occurrence of cells in which a light-induced current (≥ 20pA) was measured (red) or not (grey), after a 20-min incubation at different concentration of BMA. **D**. Current amplitudes obtained under 440nm light were compared with the IpH5 measured in the same cell, for incubation conditions containing 10μM, 20μM or 40μM BMA. Linear regression analysis did not show any significant correlation between the current amplitude induced by pH5 and by 440nm light. **E**, Light-induced current amplitude, normalized to IpH5 in the same cell, obtained with extracellular solutions containing Na^+^ or NMDG^+^. The light stimulation in the NMDG^+^ solution was carried out between the two indicated stimulations in the Na^+^ solution; the stimulation interval was 1min. *, p<0.05 between indicated conditions (one-way ANOVA with Sidak’s *post-hoc* test).

It was then tested how the conditions of the BMA incubation affected the amplitude of the light-induced current of I428C. Varying the duration of the incubation with 10μM BMA between 12 and 20 min did not affect the current amplitude (Fig 2A). The current amplitude did not depend on the BMA concentration, when tested on an incubation duration of 20 min (Fig 2B). In many experiments, the relative amplitude of the light-induced current was very low (Fig 2A and 2B). Light-induced currents were found in only ~20–40% of the measured cells (Fig 2C; cells with light-induced current amplitude >20pA were considered as expressing a light-induced current). The low current amplitudes and low fraction of positive cells after incubation with 40mM BMA may be due to a stress of the cells induced by the high BMA concentration. Fig 2D plots the I_440nm-BMA_ as a function of the I_pH5.0_ for all measured cells, showing that there was no significant correlation between the pH- and the light-induced current amplitudes. This suggests that the light-induced current amplitude depended on additional factors.

The transient H^+^-activated ASIC currents are Na^+^-selective, and ASICs are not permeable to the large cation NMDG. To further confirm that the light-induced current is mediated by the expressed ASIC1a channels, the main monovalent cation of the extracellular solution, Na^+^, was replaced with NMDG, resulting in a reversible loss of 440nm light-induced current (Fig 2E, n = 7, One-way ANOVA, Sidak’s *post-hoc* test). If ASIC1a-I428C was repeatedly activated by 440 nm light, the current amplitude was increased at the second application relative to the first and showed then a rundown. This rundown did not depend on the interval between the applications of the light pulse (S2B and S2C Fig, n = 3–6, Mixed effect One-way ANOVA, Dunnett *post-hoc* test).

### Absence of BMA-mediated light-activated currents in mutants of different ASIC1a domains

**Fig 3:**
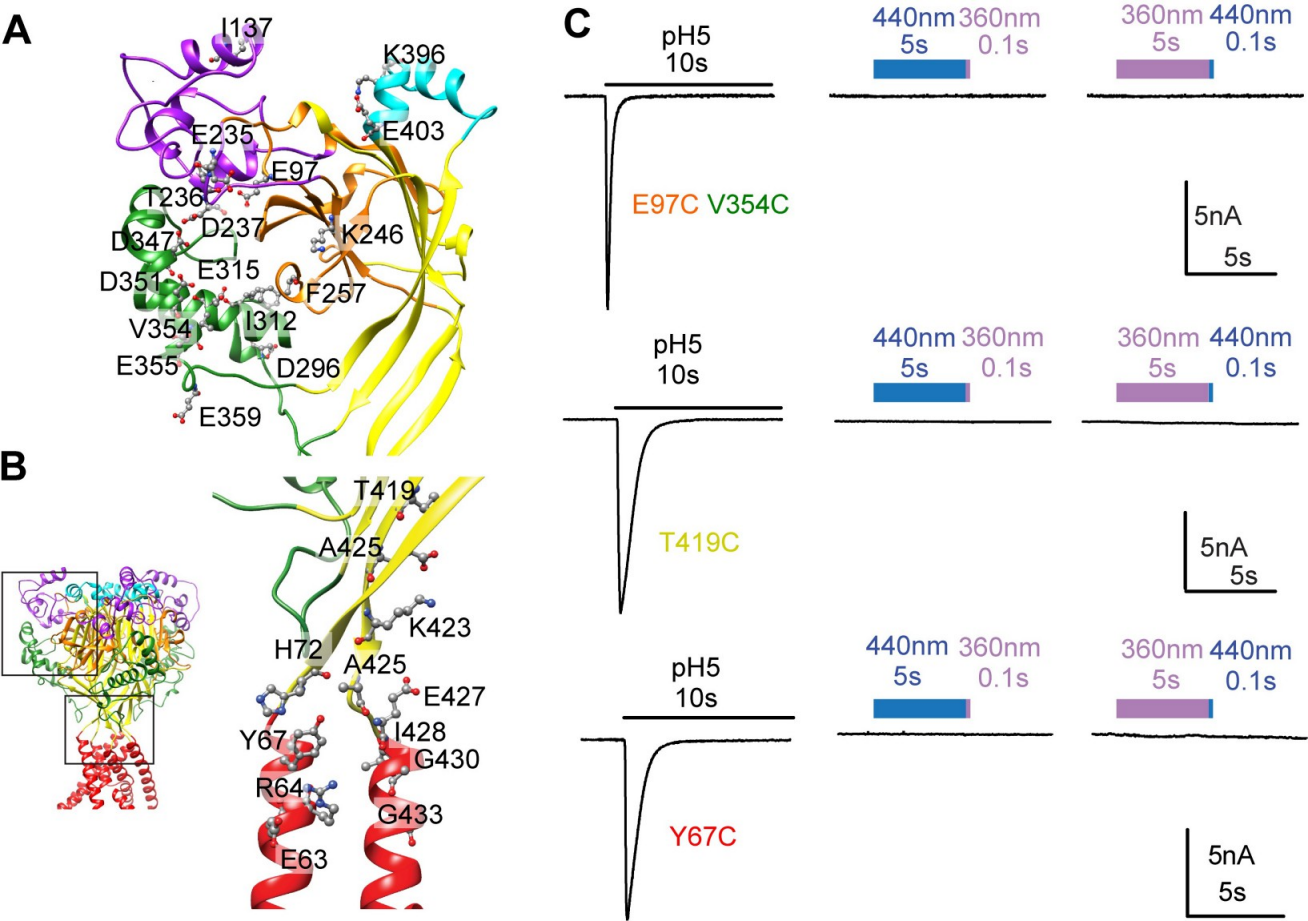
Absence of light- and BMA-activated currents in two series of ASIC1a Cys mutants. **A,** Indication of the location of Cys mutations introduced in the thumb, finger, knuckle, palm and β-ball domains. These mutations were paired to double mutants, as indicated in Table 1. **B**, Indication of the location of single Cys mutations in the transmembrane domains and the wrist. The black frames in the structural image on the left indicates the positions of the zoomed views in **A** and **B**. Current traces were obtained from whole-cell patch-clamp recordings of transfected CHO cells, voltage-clamped to -60mV. **C,** Representative traces showing a pH5-induced current and the absence of light-induced currents in E97C/V354C (top), T419C (center) and Y67C (bottom). Blue bars represent the application of 440nm light, and purple bars that of 360nm light. The color of the mutant label matches that of the domain in which the mutation is located.

Target residues for mutagenesis to Cys and docking of BMA were chosen in the extracellular pore entry and upper half of the transmembrane domains, and in extracellular domains, mostly the acidic pocket. To allow reaction of BMA with its both ends to engineered Cys residues of the channel, mutations were chosen based on structural information, in a way that the distance between the b-carbon atoms (a for Gly residues) of two residues to be replaced by Cys, either in the same or in adjacent subunits, matched approximately the length of BMA in the *cis* or *trans* conformation. This distance was further compared in structural models of the closed, open and desensitized state, with the aim of selecting mutants in which it changed between the functional states. The localization of residues that were part of selected double mutants with matching intrasubunit distances is shown in Fig 3A and that of single mutants, for which intersubunit distances matched the length of BMA, are shown in Fig 3B, and the distances are listed in Table 1. When these mutants were expressed in CHO cells, they produced normal transient H^+^-induced currents. It was then determined whether light could activate these mutants after exposure to BMA. All mutants listed in Table 1 were exposed to 440nm and 360nm light in the two timely orders, as illustrated in Fig 3C for the double mutant E97C/V354C of the acidic pocket, and the single mutants T419C of the lower palm and Y67C of the wrist. However, none of the mutants except for I428C produced light-induced currents that exceeded an amplitude of 20pA.

### Modulation of pH-induced currents by light

**Fig 4:**
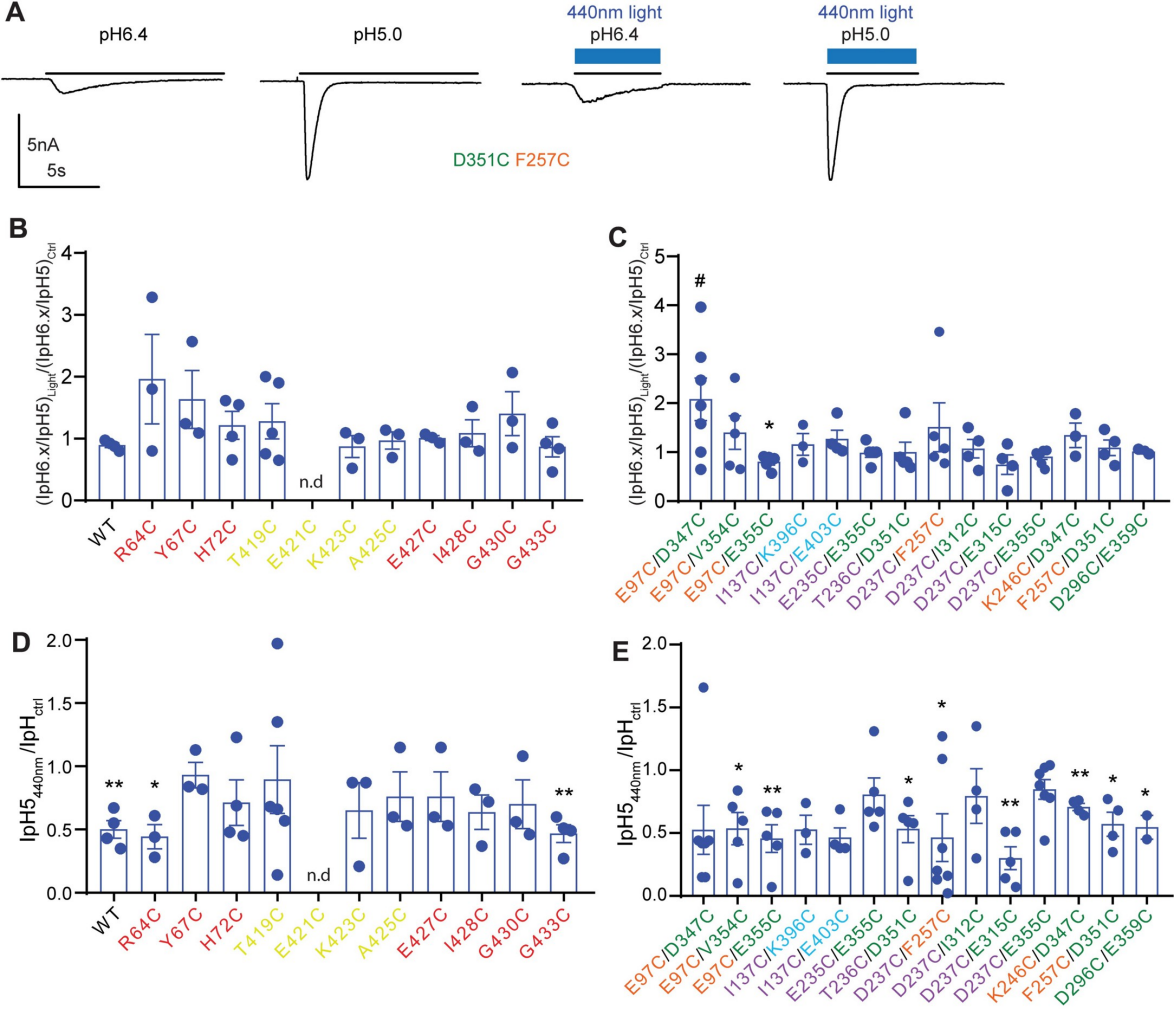
Modulatory effect of BMA and 440nm light. Traces and data were obtained from whole-cell patch-clamp recording of transfected CHO cells, voltage-clamped to -60mV. **A,** Representative traces of D351C/F257C currents induced by acidification to pH6.4 or pH5.0 from a conditioning pH of 7.4, in the absence and presence of 440nm light. The light exposure is indicated by the blue bars, the exposure to the acidic solution is indicated by the black horizontal line. **B-C**, (IpH6.x/IpH5)_Light_/(IpH6.x/IpH5)_Ctrl_ ratio in WT and mutants of the lower palm, TM1 and TM2 domain (**B**), and in the double mutants of the extracellular domain (**C**). The pH6.x corresponded to 6.0 in D237C/I312C and F257C/D351C, pH5.5 in K246C/D347C, and pH6.4. in all other mutants. **D-E**, IpH5_Light_/IpH5_Ctrl_ ratio, in WT and mutants of the lower palm, TM1 and TM2 domain (**D**), and in the double mutants of the extracellular domain (**E**). The 440nm light was pre-applied 200ms before, and co-applied during the perfusion with the acidic solutions. Statistically significant differences between 440nm and control condition are reported for IpH6.x/IpH5 and IpH5 as *, p < 0.05, **, p < 0.01 (paired t-test), and for (IpH6.x/IpH5)_440nm_/(IpH6.x/IpH5)_Ctrl_ between mutant and WT as #, p < 0.05 (One-way ANOVA with Dunnett’s *post-hoc* test). The color of the mutant label matches that of the domain in which the mutation is located.

Since light-induced currents were not observed in any mutant other than I428C, it was tested whether simultaneous application of light and low pH to BMA-exposed mutants changed the current response compared to low pH alone. The IpH6.x/IpH5 ratio (with pH6.x being a pH that induced 30–60% of the maximal current amplitude in each mutant) was measured in the absence and presence of 440nm light to determine if the pH dependence of activation was affected by BMA binding and subsequent illumination. Representative traces of pH-induced currents of D351C/F257C in the absence and presence of 440nm light are shown in Fig 4A. The ratios of the IpH6.x/IpH5 ratios obtained in the presence and absence of 440nm light, (IpH6.x/IpH5)_Light_/(IpH6.x/IpH5)_Ctrl_ are plotted and compared to that of the WT in Fig 4B and 4C, confirming the absence of a difference to the WT (One-way ANOVA, Dunnett’s *post-hoc* test) in all mutants except for E97C/D347C. The ratio of the peak amplitudes obtained at saturating pH, IpH5_440nm_/IpH5_ctrl_, indicated a decrease of the IpH5 amplitude in the WT and in many mutants (Fig 4D and 4E; paired t-test). This decrease was in none of the mutants different from the WT (Ordinary one-way ANOVA, Dunnett’s *post-hoc* test), indicating that this was a non-specific effect of 440nm light.

**Fig 5:**
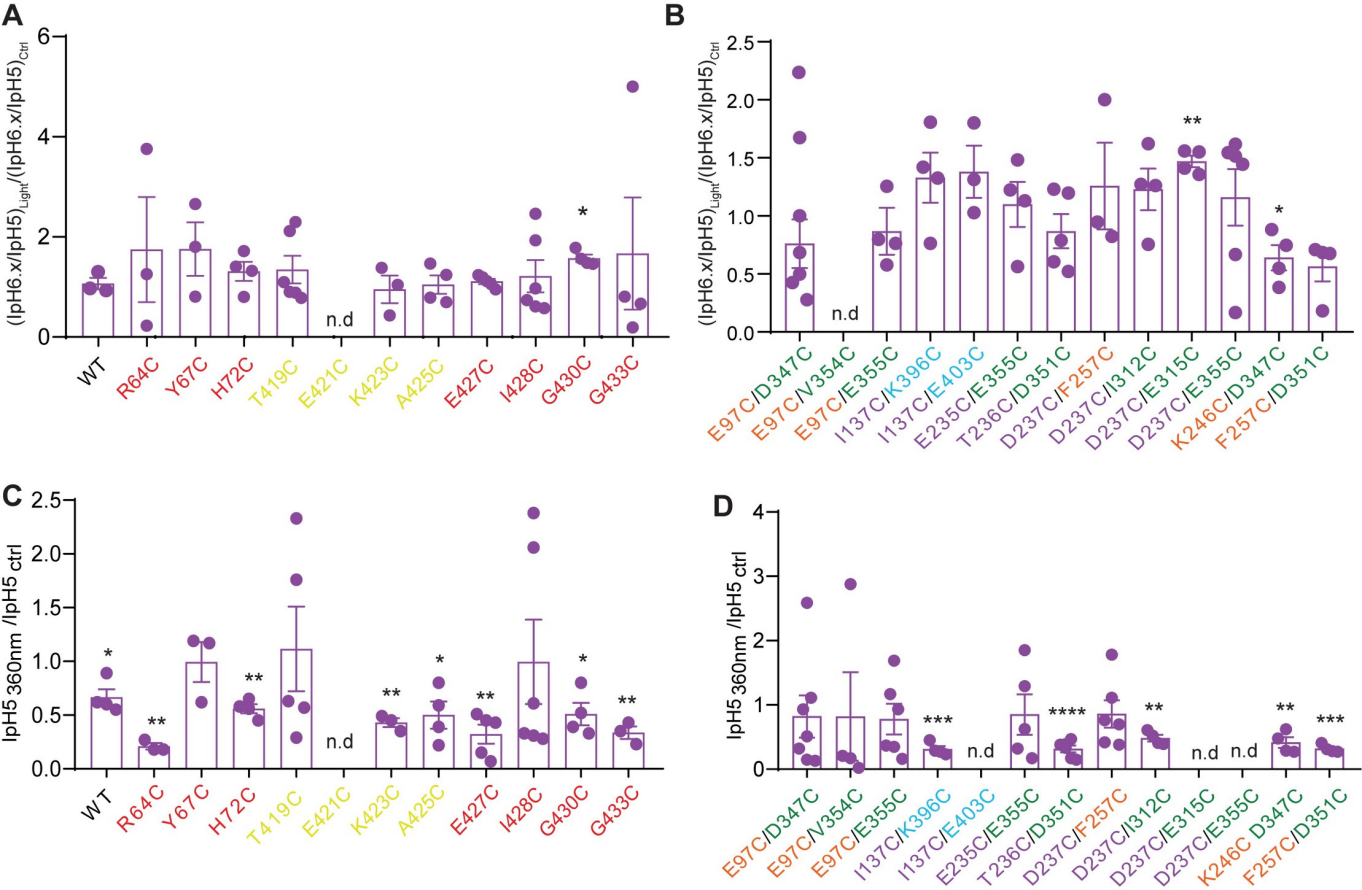
Modulatory effect of BMA and 360nm light. Data are from whole-cell patch-clamp recordings of transfected CHO cells, voltage-clamped to -60mV. **A-B**, (IpH6.x/IpH5)_Light_)/(IpH6.x/IpH5)_Ctrl_ ratio in WT and mutants of the lower palm, TM1 and TM2 domain (**A**), and in double mutants of the extracellular domain (**B**). The pH6.x corresponded to 6.0 in D237C/I312C and F257C/D351C, pH5.5 in K246C/D347C, and pH6.4. in all other mutants. **C-D**, IpH5_Light_/IpH5_Ctrl_ ratio, in WT and mutants of the lower palm, TM1 and TM2 domain (**C**), and in the double mutants of the extracellular domain (**D**). The 360nm light was pre-applied 200ms before and co-applied during the perfusion with the acidic solutions. Statistically significant differences between 360nm light and control condition are reported for IpH6.x/IpH5 and IpH5 as *, p < 0.05, **, p < 0.01, ***, p < 0.001, ****, p < 0.0001 (paired t-test). The color of the mutant label matches that of the domain in which the mutation is located.

The same measurements as described above for 440nm light were then carried out with 360nm light (Fig 5). The application of 360nm light produced on BMA-treated cells a modulatory effect on mutants G430C ((IpH6.x/IpH5)_light_/(IpH6.x/IpH5)_Ctrl_ = 1.57±0.07, n = 4, p = 0.012), D237C/E315C (1.47±0.05, n = 4, p<0.0068) and K246C/D347C (0.64±0.11, n = 4, p = 0.050; paired t-test). The (IpH6.x/IpH5)_light_/(IpH6.x/IpH5)_Ctrl_ ratio was however for none of the mutants different from that of WT (Fig 5A and 5B, ordinary one-way ANOVA, Dunnett’s *post-hoc* test). The IpH5 amplitude was significantly reduced by 360nm light in WT and several mutants (Fig 5C and 5D, paired t-test). The IpH5_light_/IpH5_Ctrl_ ratio was however not different between the WT and any of the mutants (One-way ANOVA, Dunnett’s *post-hoc* test).

### No evidence for BMA cross-linking of the I428C mutant

**Fig 6:**
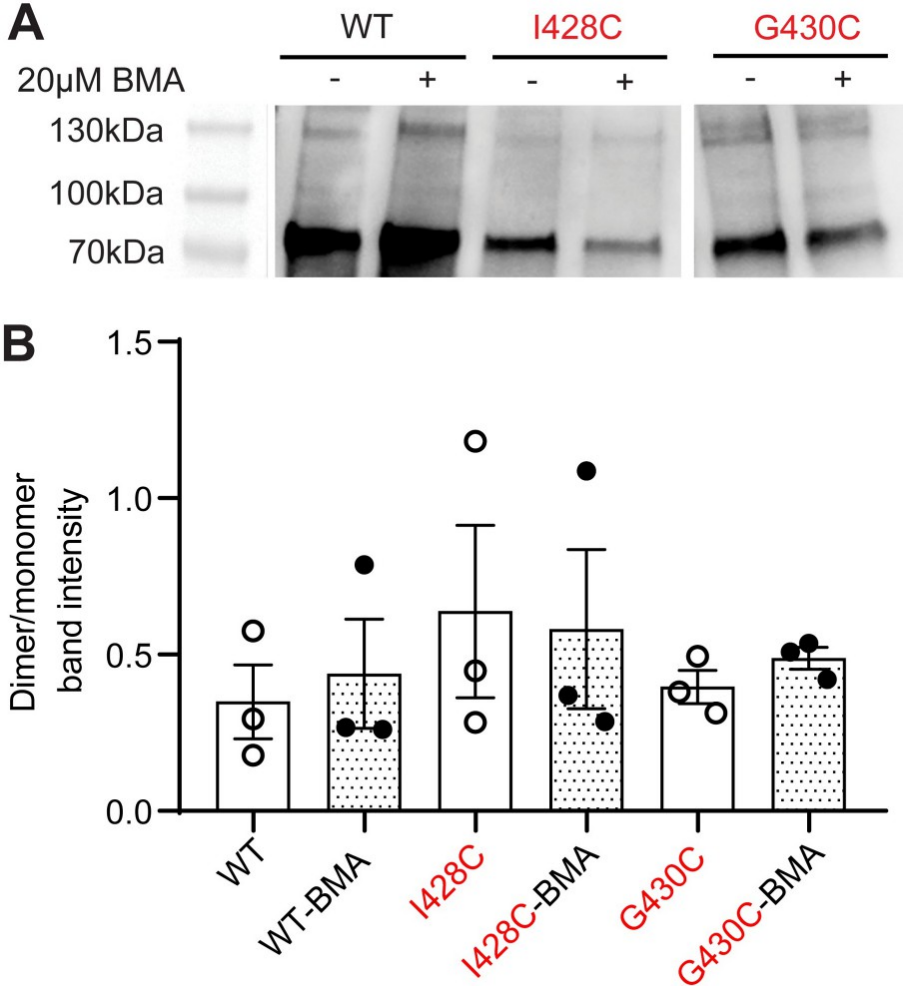
No evidence in western blot analysis for intersubunit cross-linking by BMA in I428C and G430C mutants. Cell surface-expressed proteins of transfected CHO cells were isolated by biotinylation, separated by SDS-PAGE and transferred to Western blot, and ASIC1a was detected by a specific antibody. **A**. Representative Western blots, showing the bands of monomeric ASIC1a at 70kDa, and the bands of the subunit dimer at 130kDa for the indicated constructs. The blot of WT and I428C originates from blot 1 (S1 Raw images), while that of G430C originates from blot 2. **B**, Ratio between dimeric to monomeric band intensity, n = 3.

BMA is a cross-linker, and it was assumed in a previous study [31] that BMA links in the ASIC1a-I428C mutant the engineered Cys428 of two adjacent subunits. Many studies have previously shown that monovalent sulfhydryl reagents can change the function of ASIC1a mutants [20, 36–38]. We have therefore tested biochemically in ASIC1a WT and the two mutants I428C and G430C, whether BMA cross-linked two subunits. As in the patch-clamp experiments, cells expressing these ASIC constructs were labeled with 20μM BMA for 20min in the dark. Cell surface proteins were labeled with biotin, and cells were lysed. The extracted biotinylated surface proteins were separated on SDS-PAGE and detected on western blot by using an ASIC1 antibody. The specificity of the antibody used had been demonstrated [33]. Monomeric ASIC1a WT or mutant bands migrated at ~70kDa and dimer bands at ~130kDa (Fig 6A). Comparison of the dimer / monomer band intensity ratio showed no significant difference between control and 20μM BMA treatment conditions (Fig 6B).

### Modulation of H+-activated currents by MTS cross-linkers

As an alternative approach to BMA, MTS cross-linkers were then used. These reagents can cross-link two Cys residues of a protein; their conformation is however in contrast to BMA not changed by light. An array of MTS cross-linkers of different lengths is commercially available. For each mutant, one or several MTS cross-linkers were selected based on their length (S1 Table) to match the distance measured between the b-carbon atoms (a for Gly) of the paired residues in at least one of the functional channel states (Table 1). The chosen reagents for each mutant are listed in the S2 Table. Successful cross-linking was expected to impose the distance of the cross-linker between the residue pair of the mutant to force the channel into the conformation that best matches the imposed distance between the two residues.

**Fig 7:**
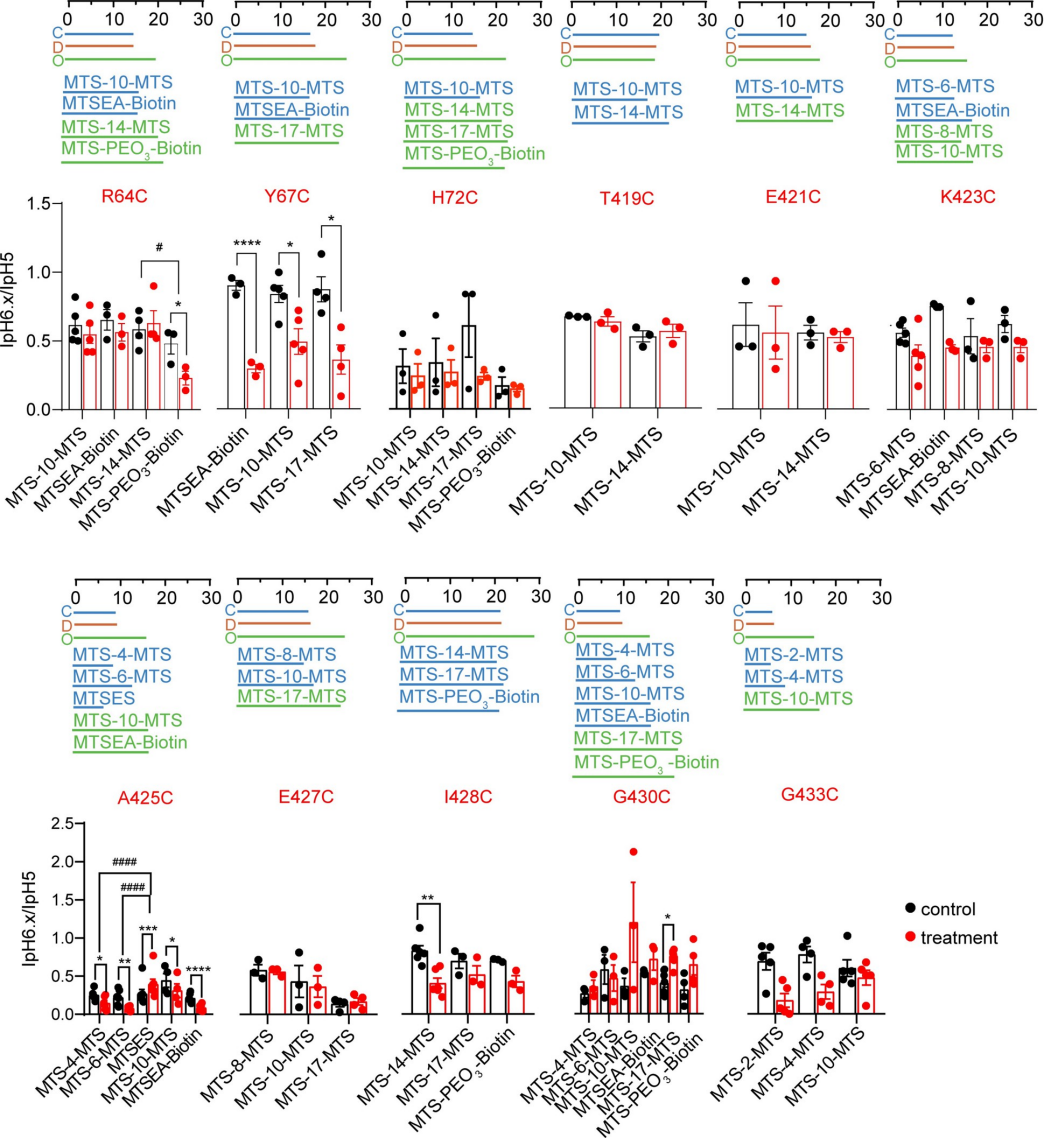
Effects of MTS reagents on the pH dependence of single mutants in the wrist and TM domains. Data were obtained from two-electrode voltage-clamp of *Xenopus* oocytes expressing the indicated mutants, clamped to -60mV. The IpH6.x/IpH5.0 ratio was determined in each cell before and after exposure to the MTS reagents and is indicated with black symbols for the control condition, and with red symbols for the condition after exposure to the MTS reagent. Changes in the IpH6.x/IpH5.0 ratio indicate that the MTS reagent affected the pH dependence. The pH6.x was pH6.6 for mutants Y67C, E427C, G430C and G433C, and pH6.4 for R64C, H72C, T419C, E421C, K423C, A425C and I428C. Differences between the control and the MTS condition are indicated as *, p < 0.05; **, p < 0.01, ***, p < 0.001 and ****, p < 0.0001 (paired t-test) and between cross-linkers and matching monovalent MTS reagents as #, p < 0.05 and ####, p < 0.0001 (Ordinary one-way ANOVA and Tukey *post-hoc* test). Above the data, a scale (in units of Å) and horizontal lines illustrate the distance between Cβ-atoms (Ca-atoms in Gly) of the mutated residues in the closed, open and desensitized state, and the length of the used MTS cross-linkers and monovalent MTS reagents used with the mutant. The color code is blue for closed, red for desensitized, and green for open; the colors of the reagents were chosen according to the best match between the length of the reagent and the distance between the two residues. The color of the mutant label matches that of the domain in which the mutation is located.

**Fig 8:**
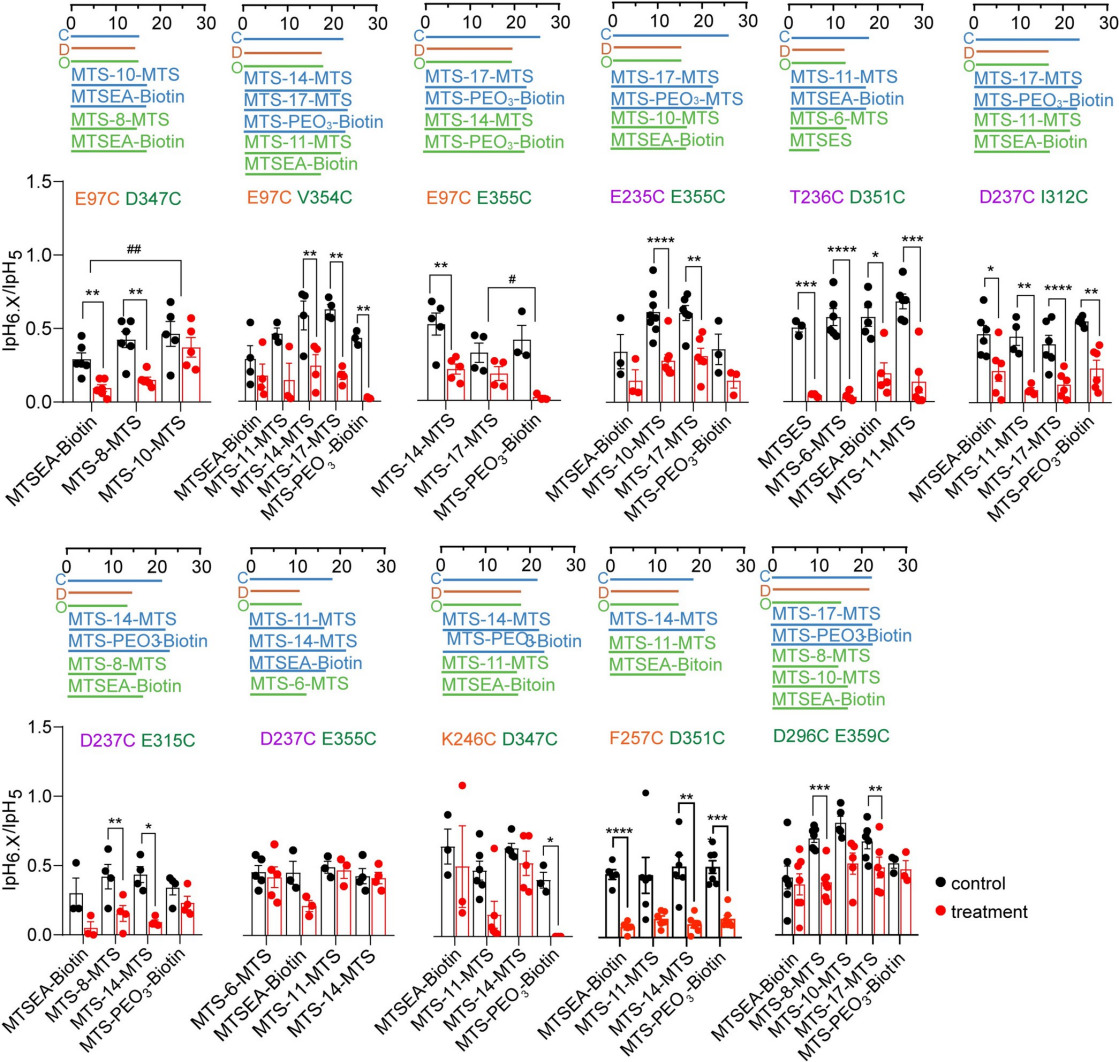
Effects of MTS reagents on the pH dependence of double mutants in the extracellular domain. Data were obtained from two-electrode voltage-clamp of *Xenopus* oocytes expressing the indicated constructs, clamped to -60mV. The IpH6.x/IpH5.0 ratio was determined in each cell before and after exposure to the MTS reagent and is indicated with black symbols for the control condition, and with red symbols for the condition after exposure to the MTS reagent. Changes in the IpH6.x/IpH5.0 ratio indicate that the MTS reagent affected the pH dependence. The pH6.x was pH6.6 for the mutant E97C-E355C, pH6.4 for E97C/V354C, E235C/E355C, D237C/E315C, E237C/E355C and D296C/E359C, pH6.2 for T236C/D351C and D237C/I312C, pH6.0 for E97C/D347C and F257C/D351C, and pH5.5 for K246C/D347C. Differences between the control and the MTS condition are indicated as *, p < 0.05; **, p < 0.01, ***, p < 0.001 and ****, p < 0.0001 (paired t-test) and between cross-linkers and matching monovalent MTS reagents as #, p < 0.05; ##, p < 0.01 (Ordinary one-way ANOVA and Sidak’s *post-hoc* test). The meaning of the colored horizontal lines above the graphs is the same as in Fig 7. The color of the mutant label matches that of the domain in which the mutation is located.

The functional analysis with MTS cross-linkers was performed on most of the mutants that had been used in the first part of this study. For each mutant, the ASIC current was measured at two different pH values before and after exposure to the cross-linker (applied for 3 min at 1mM), pH6.x that induced 30–60% of the maximal current amplitude under control conditions, and pH5 for the maximal current amplitude. The current amplitude induced by pH5, and the IpH6.x/IpH5 ratio were then compared between the control and the cross-link condition. Since monovalent MTS reagents can also affect the function of ASIC1a Cys mutants [36, 37, 39, 40], an observed effect of a cross-linker may also be induced if the reagent reacts only at one of its ends with a Cys residue of the channel. It was assumed that an effect due to such a mechanism should be similar to that of a monovalent MTS reagent of similar size. If an MTS cross-linker affected the function of a given mutant, the experiment was therefore repeated with a monovalent MTS reagent of similar size. In Figs 7 and 8, the Cys-Cys distance is indicated for each mutant in the closed, open and desensitized state by horizontal, colored lines, and for comparison, the lengths of the used reagents are shown in matching colors. For many mutants, the MTS reagents induced an acidic shift of the pH dependence, as indicated by a decrease of the IpH6.x/IpH5 ratio (Figs 7 and 8). If the effect of the cross-linker was greater than that of the monovalent compound, it suggested that the difference may be due to the cross-linking of two Cys residues. The (IpH6.x/IpH5)_MTS_/(IpH6.x/IpH5)_Ctrl_ ratio with MTS cross-linkers showed a significantly stronger change than with a size-matched monovalent MTS reagent in A425C (MTS-4-MTS and MTS-6-MTS compared to MTSES, Fig 7) and E97C-E355C (MTS-17-MTS compared to MTS-PEO_3_-Biotin, Fig 8; ANOVA, followed by Tukey *post-hoc* test). Several mutants showed tendencies of stronger effects on the (IpH6.x/IpH5)_MTS_/(IpH6.x/IpH5)_Ctrl_ ratio with bifunctional as compared to monovalent reagents, T236C-D351C (MTS-11-MTS compared to MTSEA-Biotin), D237C-I312C (MTS-17-MTS compared to MTS-PEO_3_-Biotin) and D237C-E315C (MTS-14-MTS compared to MTS-PEO_3_-Biotin, Fig 8). In control experiments, the (IpH6.4/IpH5)_MTS_/(IpH6.4/IpH5)_Ctrl_ ratio was determined with ASIC1a WT for several compounds, showing no statistically significant effect (S4 Fig).

**Fig 9:**
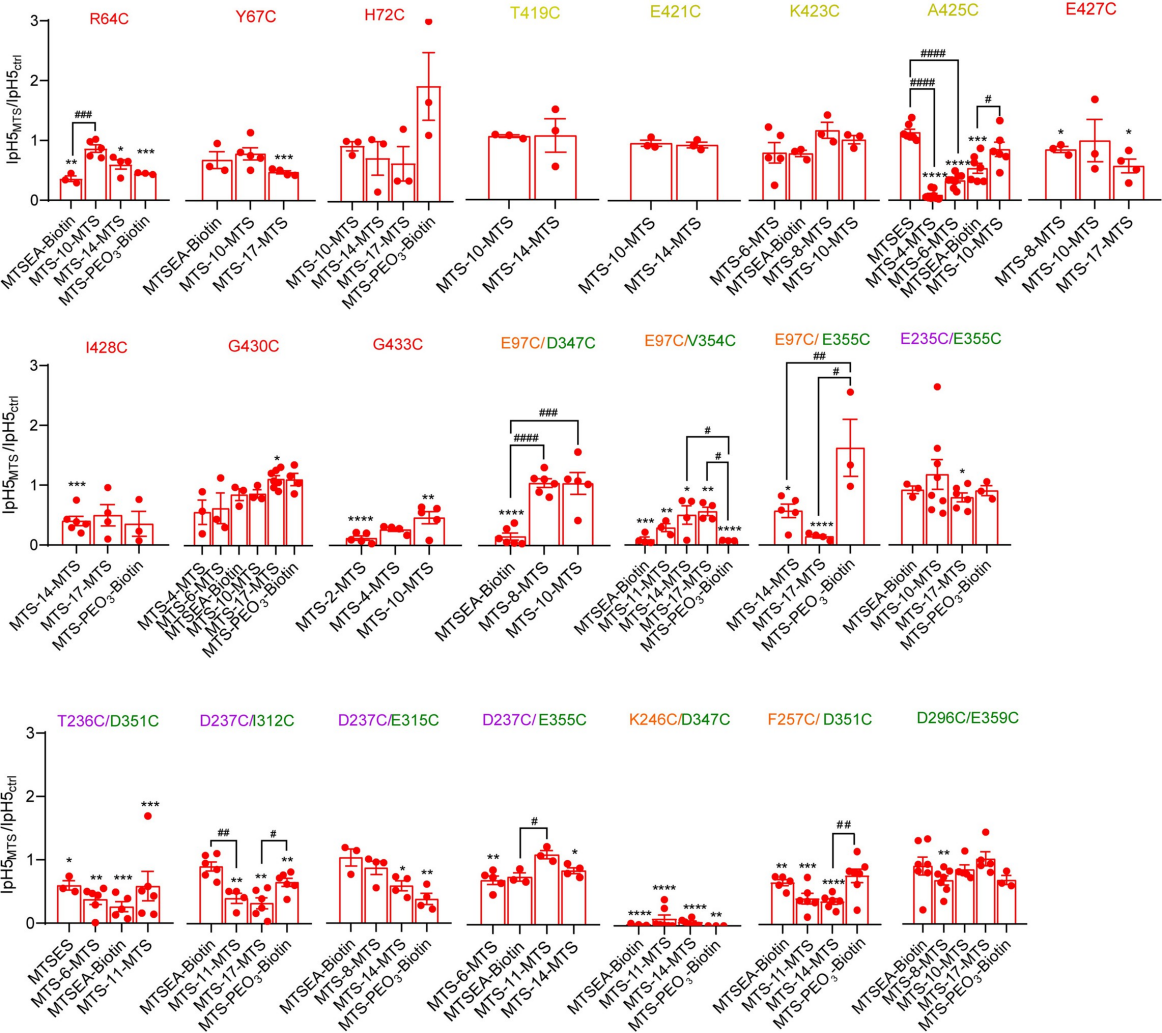
Changes in maximal peak current amplitude induced by MTS reagents. IpH5_MTS_/IpH5_Ctrl_ ratios plotted here were determined in the experiments described in Figs 7 and 8. Differences between the control and the MTS condition are indicated as *, p < 0.05; **, p < 0.01, ***, p < 0.001 and ****, p < 0.0001 (paired t-test), and between cross-linkers and matching monovalent MTS reagents as #, p < 0.05; ##, p < 0.01, ###, p < 0.001 and ####, p < 0.0001 (Ordinary One-way ANOVA and Tukey *post-hoc* test). The color of the mutant label matches that of the domain in which the mutation is located.

From the same experiments, the effect of the MTS reagents on the IpH5 was analyzed. In many mutants, a significant decrease in IpH5 was observed, which was however in most cases not stronger than the effect of the corresponding monovalent MTS reagent (Fig 9). Exceptions, with stronger effects of the cross-linkers on IpH5, were the mutants A425C, E97C-E355C, D237C-I312C and F257C-D351C.

### Stronger cross-linker-induced shift of pH dependence in D237C/I312C than in the corresponding single mutants

**Fig 10:**
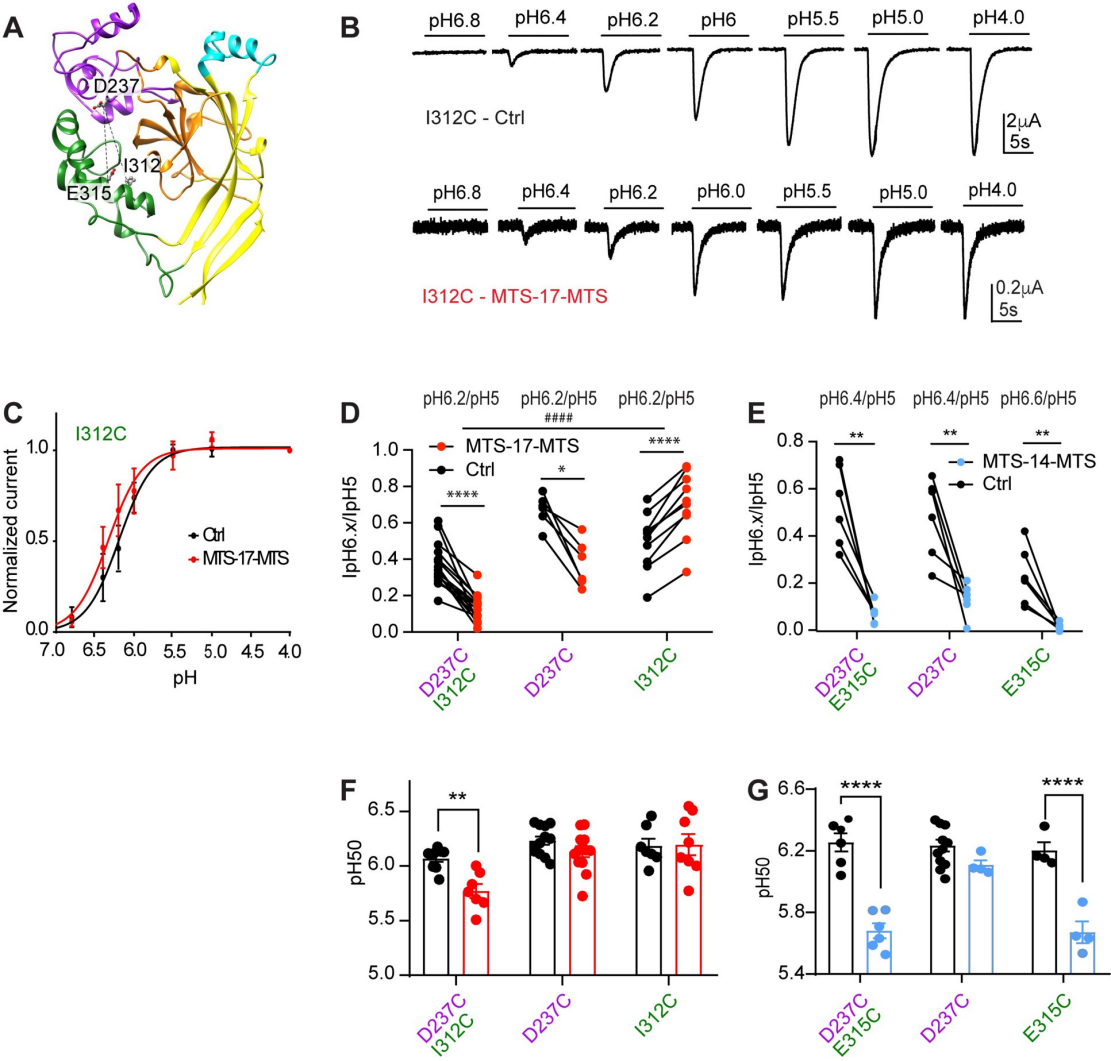
Effect of MTS-cross-linkers on the pH dependence of selected double mutants and corresponding single mutants. Data were obtained from two-electrode voltage-clamp of *Xenopus* oocytes expressing the indicated constructs, clamped to -60mV. **A,** Structural representation of hASIC1a model in the closed state, identifying the paired residues of double mutants by the connecting dotted lines. **B**, Current traces of representative experiments determining the pH dependence of I312C under control conditions (upper panel) and after treatment with MTS-17-MTS (bottom). **C,** pH-response curves of I312C obtained under control conditions (ctrl, black), and after exposure to MTS-17-MTS (red; n = 12–13). **D-G**, black symbols refer to control conditions, colored symbols to MTS exposure as indicated. **D-E**, IpH6.x/IpH5 ratio under control conditions or after MTS exposure, for double and corresponding single mutants, (**D)**, D237C/I312C, (**E**), D237C/E315C. **F-G**, pH of half-maximal activation (pH_50_) values, obtained under control conditions or MTS reagent exposure; significantly different between Ctrl and MTS condition, *, p< 0.05; **, p < 0.01, ***, p < 0.001 for **D-E** (paired t-test) and **F-G** (unpaired t-test). ####, p < 0.0001; different between single and corresponding double mutant (Ordinary one-way ANOVA, Tukey post hoc test). The color of the mutant label matches that of the domain in which the mutation is located.

For two promising double mutants (Fig 10A), the IpH6.x/IpH5 ratio was determined, and the pH dependence of activation was measured under control conditions and after exposure to the MTS cross-linker, in the double, and also in the corresponding single mutants. The measurement of the pH dependence of activation, as illustrated in Fig 10B and 10C for I312C, yields the midpoint of activation, the pH_50_ value. The ratio of IpH6.x/IpH5 and the pH_50_ of activation are plotted for the two double mutants and the corresponding single mutants in Fig 10D–10G. If cross-linking was responsible for the functional effect of a cross-linker, we would expect small effects in the two single mutants, and a larger effect in the double mutant. For the D237C/E315C mutant, exposure to MTS-14-MTS decreased the IpH6.4/IpH5.0 ratio of D237C but did not change the pH50 of this mutant. On D237C/E315C and E315C, the IpH6.x/IpH5.0 ratio and pH50 values were decreased in a very similar way (Figs 10E and 10G and S5B and S5D), suggesting together that the observed functional changes in D237C/E315C are to a large extent due to the effects of the reaction with the mutation E315C. The IpH6.2/IpH5 ratio change induced by MTS-17-MTS was opposite between D237C/I312C and I312C, and this ratio showed a tendency of being lower in D237C/I312C than in D237C (p = 0.0714; Figs 10D and S5A). The pH50 was changed significantly by MTS-17-MTS in D237C/I312C, but not in the corresponding single mutants (Fig 10F). The IpH5 was not affected by MTS-17-MTS in I312C, increased in D237C, and decreased in D237C/I312C (S5C and S5D Fig). The above data suggest that a cross-link between D237C and I312C by MTS-17-MTS causes the observed acidic shift of the pH dependence. Such a cross-linking between D237C and I312C would position these two residues in a relative distance that is similar to that observed in the closed state.

## Discussion

In this study we used cross-linking compounds on ASIC1a to detect conformational changes involved in channel activation. We show that ASIC1a-I428C that has reacted with the optical tweezer BMA can be activated by light, and that this does not involve a cross-linking between I428C of adjacent subunits. None of the other tested mutants containing single or double Cys mutations in the pore, wrist or extracellular domains were activated by light after exposure to BMA. MTS cross-linkers and monovalent MTS reagents changed the pH dependence of many of these mutants. In the A425C mutant, short cross-linkers had stronger effects on pH dependence and maximal current amplitude than a size-matched monovalent MTS reagent. Analysis of the pH dependence of activation of two selected double mutants and the corresponding single mutants showed that MTS-17-MTS produced an acidic shift in the double mutant D237C/I312C of the acidic pocket, and that this shift was smaller in the single mutant D237C, and absent in I312C. The length of this compound corresponds to the distance between the two engineered Cys residues of D237C/I312C in the closed state and suggests therefore that constraining the distance between these two residues may hinder ASIC activation.

### Previous findings with MTS reagents in the extracellular domain of ASIC1a

For the mutant hASIC1a-G430C it had previously been observed that monovalent MTS reagents such as MTSPTrEA produced an alkaline shift in the pH dependence of activation, increasing therefore the sensitivity for activation [36]. MTSET and MTSPTrEA induced in this mutant a current at pH7.4 in the absence of extracellular acidification (S3 Table) [36]. The homologous mutation in mouse ASIC1a, G428C, was also shown to be involved in channel activation (S3 Table) [37]. For Gly430, the intersubunit distances are 16.6 Å in the open, and ~9.5 Å in the closed and desensitized states. In our hand, MTS-4-MTS and MTS-6-MTS that are close to the intersubunit distance between Gly430 residues in the closed state, did not change the IpH6.6/IpH5 ratio. The larger reagent MTS-17-MTS (length 23 Å) induced an alkaline shift of the pH dependence, which was however not different from that induced by the size-matched control compound MTS-PEO_3_-Biotin. This observed alkaline shift was therefore most likely due to a steric effect, and not to cross-linking. For two other mutants, hASIC1a-E315C and -D347C, it had previously been shown that MTSET shifted the pH_50_ of activation to more acidic values and inhibited the peak current amplitude [28].

### Light-dependent activation of ASIC1a after exposure to BMA

To date, only a few publications have documented activation of ion channels by optical tweezers. This approach was in several studies successful with P2X receptors [31, 32, 41]. One of these studies also showed that two ASIC1a mutants, I428C and G430C, were opened by light after reaction with BMA [31]. In our hands, I428C, but not G430C produced light-induced channel activation after exposure to BMA. Comparison of the distances in the structures indicated intersubunit distances between the engineered Cys residues that appeared to be too long in the case of I428C and too short in the case of G430C for gating by BMA (Table 1). Our biochemical analysis of cell surface-expressed ASIC1a did not show cross-linking of two subunits in any of these mutants by BMA. BMA-modified I428C likely contains attached BMA molecules without the occurrence of a cross-linking between subunits, and application of 440nm light may change the orientation of the BMA chain to open the channel in a similar way as MTSET and MTS-PTrEA open G430C [36].

### Modulatory effect of BMA by application of light on hASIC1a activity

For this study we had selected mutations in the wrist and pore, and pairs of mutations in other extracellular channel domains, for which the distance between the mutated residues in structural models of at least one of the functional states matched the end-to-end distance of BMA in the *cis* or *trans* conformation (Table 1). In all the mutants shown in Table 1 except for I428C, application of 440nm and 360nm light after exposure to BMA did not induce any light-induced current. Comparison of the (IpH6.x/IpH5)_440nm_/(IpH6.x/IpH5)_Ctrl_ ratio showed a significant difference from the WT only for E97C/D347C, located in the acidic pocket. Analysis of the change in the IpH6.x/IpH5 ratio under exposure to 360nm indicated a significant difference between control and light condition in G430C, D237C/E315C and K246C/D347C. Gly430 is part of the wrist, and the residues mutated in the two double mutants are located in the acidic pocket (Fig 3A and 3B). 360nm light induced an alkaline shift in the pH dependence of G430C. Since BMA exposure did not cross-link G430C residues of neighboring subunits, it is likely that the attachment by one end of the BMA molecules to the G430C of one or several subunits per trimer, and the light-induced change in BMA conformation led to the shift in pH dependence, as have done monovalent MTS reagents in a previous study [36], and as we observed here in experiments with *Xenopus* oocytes. Application of 360nm light brings BMA in the *cis* conformation [31]. Its length in this conformation (~13Å) would correspond in the double mutant D237C/E315C to the open or desensitized state distance between the two engineered Cys residues. 360nm light induced an alkaline shift of the D237C/E315C pH dependence, as expected for a constraint that favors the open state (Fig 5B). The K246-D347 distance is shorter in open and desensitized conformations as compared to the closed state. The deduced acidic shift in K246C/D347C (Fig 5B) is inconsistent with the higher distance in the closed state.

### Functional analysis of hASIC1a mutants using MTS cross-linkers

Thiol-specific MTS cross-linkers with various lengths of spacer arms were selected to match the distance between the Cβ [Cα for Gly] of residue pairs located in ASIC domains that have been associated with activation. Several studies have previously used monovalent MTS reagents to analyze conformational changes associated with ASIC1a functional states [26, 36, 37, 39, 40, 42]. To date, only very few studies have used MTS cross-linkers [41, 43, 44]. Loo and Clarke used different thiol-specific MTS cross-linkers to assess the drug-binding domain of P-glycoprotein and the molecular mechanism associated to ATP-hydrolysis that causes drug transport [43]. Fryatt et al. investigated the conformation of intracellular regions of the human P2X1 receptor [41]. Cross-linking in the TM domain inhibited the ATPase activity due to hindered conformation changes. A study with ENaC, which belongs to the same family as ASICs, investigated the involvement of intersubunit distance changes in channel activity, by using MTS cross-linkers [44]. Cross-linking at the level of the lower palm and thumb increased the ENaC activity if the cross-linker was relatively long.

Our analysis with ASIC1a showed in some mutants a stronger change in the (IpH6.x/IpH5)_MTS_/(IpH6.x/IpH5)_Ctrl_ ratio after exposure to MTS cross-linkers in comparison to monovalent MTS reagents. The residue Ala425 is located at the extracellular pore entry, with intersubunit distances of ~16Å in the open, and ~9Å in the desensitized and closed structures. Small MTS cross-linkers (< 13Å) strongly decreased the pH5-induced current amplitude and the IpH6.4/IpH5.0 ratio, while the size-matched monovalent MTS reagent MTSES did not affect the IpH5 and increased slightly the IpH6.4/IpH5.0 ratio. This suggests that MTS-4-MTS and MTS-6-MTS cross-linked A425C residues of neighboring subunits, possibly by forcing a distance that is closer to that seen in the non-conducting states.

Besides A425C, MTS cross-linkers affected the IpH5 or the IpH6.x/IpH5 ratio of several double mutants. In F257C/D351C, monovalent MTS reagents and cross-linkers induced a similar acidic shift of the pH dependence. A IpH5 decrease, as induced by cross-linkers on this mutant, was not observed after exposure to the matched monovalent MTS reagent. In the D237C/E315C mutant, in which 360nm light after BMA exposure had induced an alkaline shift of the pH dependence (Fig 5B), the exposure to MTS-8-MTS and MTS-14-MTS induced a strong decrease in the IpH6.4/IpH5 ratio—thus an acidic shift—which was however not significantly different from the effects of size-matched monovalent MTS reagents (Fig 8). Analysis of the corresponding single mutants (Fig 10E and 10G) showed that these effects were to a large extent due to the E315C mutation. In the mutant D237C/I312C, the cross-linker MTS-17-MTS, whose length matches the closed state distance between the two mutated residues, produced an acidic shift of the activation pH dependence, while it induced a smaller acidic shift in D237C, and had no effect on I312C. A cross-linking with MTS-17-MTS of D237C and I312C would lock the acidic pocket in its closed state conformation. Our experiments do however not prove the formation of a cross-link between D237C and I312C.

### Rare occurrence of cross-linking in single and double mutants

Despite of testing 11 single mutants with intersubunit Cys-Cys distances matching the length of BMA and MTS crosslinkers, and of 11–14 double mutants in which intrasubunit Cys-Cys distances were compatible with the length of the crosslinkers, we found that for most mutants no cross-linking occurred as judged by the absence of functional effects. In some mutants, cross-linking may have occurred but did not change channel function. In a recent study with the trimeric P2X receptor, it was found that of 10 tested single mutants, exposure to the BMA analog 4,4´-bis(maleimido-glycine) azobenzene resulted in light-induced currents in 6 mutants, and that in 3 of these mutants, the reagent cross-linked adjacent subunits [32]. The reason for the very low cross-linking success rate in our study is not clear. It is possible that once the BMA has been attached at one end, the orientation of the molecule or steric constraints due to the conformation of the channel protein may make it difficult to react with the second Cys residue.

### MTS reagents affect the function of wrist and acidic pocket mutants

Although this study did not identify many functional changes that were due to cross-linking, it showed that channels carrying engineered Cys residues at many different positions were modulated by the reaction with MTS compounds, including the following single mutants in the pore entry and wrist: Arg64, Tyr67, Ala425, Glu427, Ile428, Gly430 and Gly433. The reagents induced in most of these mutants acidic shifts in pH dependence and a decrease of the maximal current amplitude. Exceptions were alkaline shifts in pH dependence of A425C by MTSES and of G430C by MTS-17-MTS. These are all positions in the upper part of the transmembrane segments or the wrist, indicating that this region is sensitive to modifications, that can, depending on the exact position, either favor or hinder channel opening. In all tested double mutants, at least one tested MTS reagent affected the pH dependence or the maximal current. This contrasted with the absence of any significant effect on WT. For the double mutants it was not always clear whether only one of the two mutations was responsible for the effect. While the observed effects were in most cases an acidic shift and a decrease of the maximal current amplitude, we found also other effects, such as a tendency of an IpH5 increase of E97C/E355C after exposure to MTS-PEO_3_-Biotin, while two cross-linkers decreased the IpH5. Stronger effects of monovalent as compared to cross-linking reagents, as observed with R64C, E97C/D347C, E97C/E355C and K246C/D347C, may be due accessibility constraints.

## Conclusion

In conclusion, two different cysteine-based cross-link approaches were applied with the aim of identifying residues and associated conformational changes involved in ASIC1a activation. The analysis revealed the induction of current by light in I428C without cross-link formation. The use of MTS cross-linkers and functional analysis suggested cross-linking of A425C residues of the wrist and between D237C and I312C of the acidic pocket with effects that were consistent with an increase of the intersubunit A425C distance and a decrease of the D237C/I312C distance upon activation.

## Supporting information

S1 Fig Light-induced current in rP2X2. **A-B**, Data are from whole-cell patch-clamp recordings of transfected HEK cells, voltage-clamped to -60mV. **A**, 7μM ATP-induced (left) and 440nm light-activated current (right) in rP2X2 P329C after exposure to BMA. The blue bar over the right trace indicates the exposure to 440nm light, the purple bar indicates exposure to 360nm light. **B**, Comparison of 7μM ATP-induced and light-induced current in rP2X2 P329C from paired experiments. In each cell, the current amplitudes were normalized to that induced by 7 mM ATP. Statistical significance was determined by paired t-test, n = 11; ****, p < 0.0001. **C**, Light intensity, measured by a portable light meter on the microscope stage, as a function of the brightness level value set in the DC2000 light controller device (left) or by adjusting the voltage input to the controller by the PatchMaster software.
(TIF)

S2 Fig Properties of I428C. **(A)** pH dependence of I428C. Normalized current response as a function of the stimulation pH for ASIC1a I428C (n = 7). Currents were induced by acidification for 5s followed by conditioning pH7.4 for 55s. Data are from whole-cell patch-clamp recordings of transfected CHO cells, voltage-clamped to -60mV. **B-C**, Light-induced current amplitudes, normalized to the amplitude in sweep number 2, with a sweep interval of 3min (**B**) and 1min (**C**) (n = 4).
(TIF)

S3 Fig Absence of light-induced currents in hASIC2a carrying Cys mutations of the residues homologous to ASIC1a-I428C and -G430C. Cells expressing mutants were labelled with 10μM BMA for 12 min prior to the measurement. Data were obtained from whole-cell patch-clamp recording of transfected CHO cells, voltage-clamped to -60mV. Channels were exposed to pH5 or to 440nm light for 5s and 360nm light for 0.1s.
(TIF)

S4 Fig Effect of MTS crosslinker reagents on WT. Data were obtained from two-electrode voltage-clamp of *Xenopus* oocytes expressing the WT ASIC1a clamped to -60mV. Currents were induced by exposure to pH6.4 and pH5.0 before and after exposure to MTS crosslinking reagents (1mM, 3min), in the same cell. The currents were normalized as indicated.
(TIF)

S5 Fig Current ratios in selected mutants. Data were obtained from two-electrode voltage-clamp of *Xenopus* oocytes expressing the indicated mutants, clamped to -60mV. Current ratios obtained from the indicated mutants after exposure to the MTS crosslinking reagent, normalized to the control measurement done before MTS reagent incubation. **A-B**, Ratio of (IpH6.x/IpH5)_MTS_/(IpH6.x/IpH5)_ctrl_ and **C-D**, Ratio of IpH5_MTS_/IpH5_ctrl_. **A, C,** D237C/I312C, **B, D,** D237C/E315C. Statistical significance was determined by Ordinary one-way ANOVA and Dunnett’s *post-hoc* test, n = 6–17; ^#^, p < 0.05, ^###^, p < 0.001, ^####^, p<0.0001. The pH conditions are the same as in Fig 10. The reagents were: With D237C/I312C and corresponding single mutants, MTS-17-MTS; With D237C/E315C and corresponding single mutants, MTS-14-MTS.
(TIF)

S1 Table Length of MTS cross-linkers and monovalent reagents. The length of each MTS cross-linker or monovalent reagent was determined as the distance between the Sulphur atoms on both ends after the release of sulfinic acid (SO_2_CH_3_; for cross-linkers), and between the Sulphur atom and the other end of the molecule (monovalent MTS reagents).
(PDF)

S2 Table Selection of MTS cross-linkers and monovalent MTS reagents for the different mutants. The indicated MTS cross-linkers were chosen for each mutant, based on the match between the cross-linker length and the distance between engineered Cys in the open (4NTW), desensitized (4NYK) and closed state (5WKU) models of human ASIC1a. Monovalent MTS reagents of similar length were chosen as controls. #, the length of the reagent matches the Cys-Cys distance of the desensitized state.
(PDF)

S3 Table Summary of functional information obtained from previous studies using monovalent MTS reagents on ASIC1a mutants. (PDF)

S1 Raw images This file shows the uncropped blots underlying the images and the analysis shown in Fig 6. (PDF)

S1 Data Listing of data, on which the figures are based. (XLSX)

## Abbreviations

ASIC: Acid-Sensing Ion Channel
cASIC1: chicken ASIC
cDNA: complementary deoxyribonucleic acid
CHO: Chinese Hamster Ovary
ASIC1a: human ASIC1a
mASIC1a: mouse ASIC1a
pH_50_: pH of half-maximal activation
WT: wild-type
BMA: 4,4′-Bis(maleimido)azobenzene
MTS: Methanethiosulfonate
