# Supplementary figures and images for: Probing conformational changes during activation of ASIC1a by an optical tweezer and by methanethiosulfonate-based cross-linkers
Source: PLoS One. 2022 Jul 8;17(7):e0270762. doi: 10.1371/journal.pone.0270762 (PMC9269482; doi:10.1371/journal.pone.0270762)

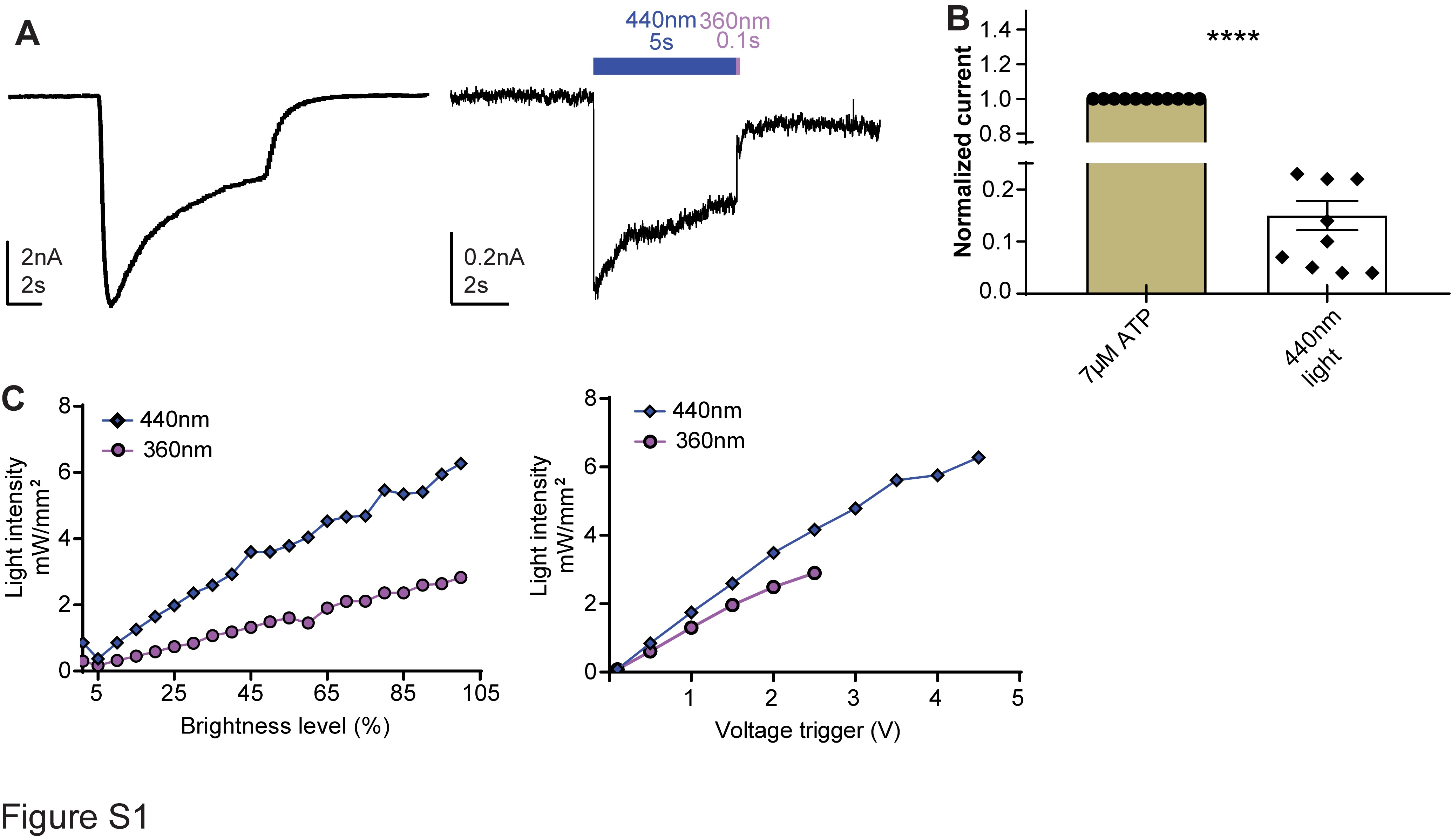

Supplement: S1 Fig — A-B, Data are from whole-cell patch-clamp recordings of transfected HEK cells, voltage-clamped to -60mV. A, 7μM ATP-induced (left) and 440nm light-activated current (right) in rP2X2 P329C after exposure to BMA. The blue bar over the right trace indicates the exposure to 440nm light, the purple bar indicates exposure to 360nm light. B, Comparison of 7μM ATP-induced and light-induced current in rP2X2 P329C from paired experiments. In each cell, the current amplitudes were normalized to that induced by 7 mM ATP. Statistical significance was determined by paired t-test, n = 11; ****, p < 0.0001. C, Light intensity, measured by a portable light meter on the microscope stage, as a function of the brightness level value set in the DC2000 light controller device (left) or by adjusting the voltage input to the controller by the PatchMaster software. (TIF) [file pone.0270762.s001.tif]

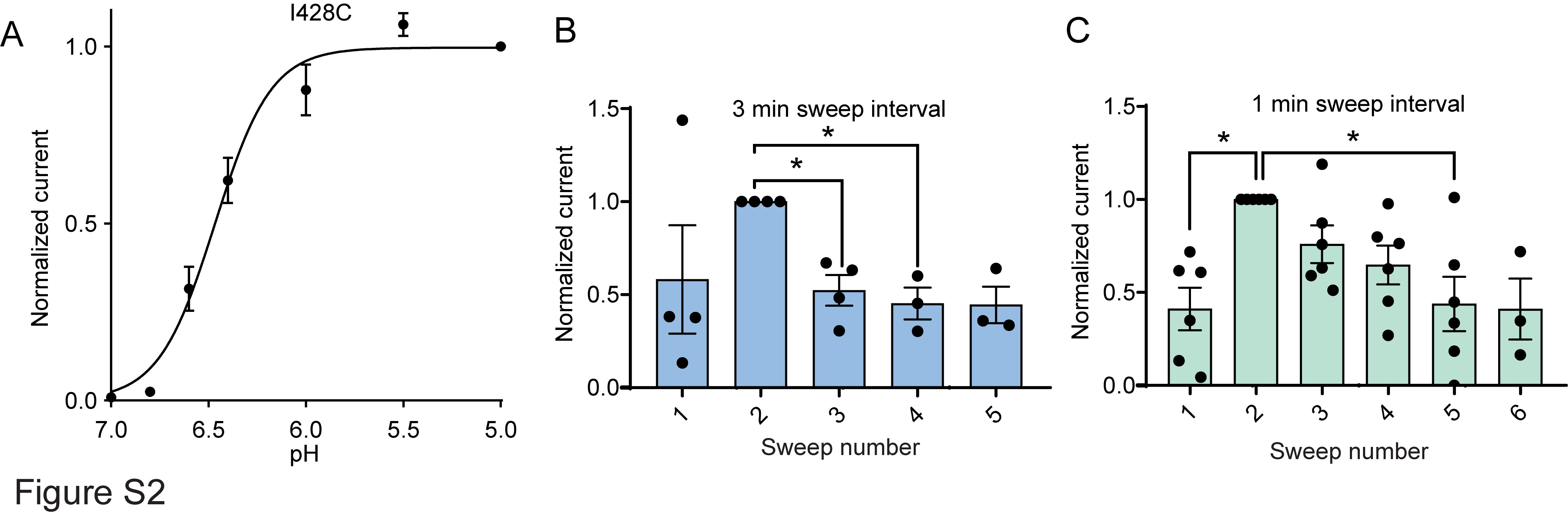

Supplement: S2 Fig — (A) pH dependence of I428C. Normalized current response as a function of the stimulation pH for ASIC1a I428C (n = 7). Currents were induced by acidification for 5s followed by conditioning pH7.4 for 55s. Data are from whole-cell patch-clamp recordings of transfected CHO cells, voltage-clamped to -60mV. B-C, Light-induced current amplitudes, normalized to the amplitude in sweep number 2, with a sweep interval of 3min (B) and 1min (C) (n = 4). (TIF) [file pone.0270762.s002.tif]

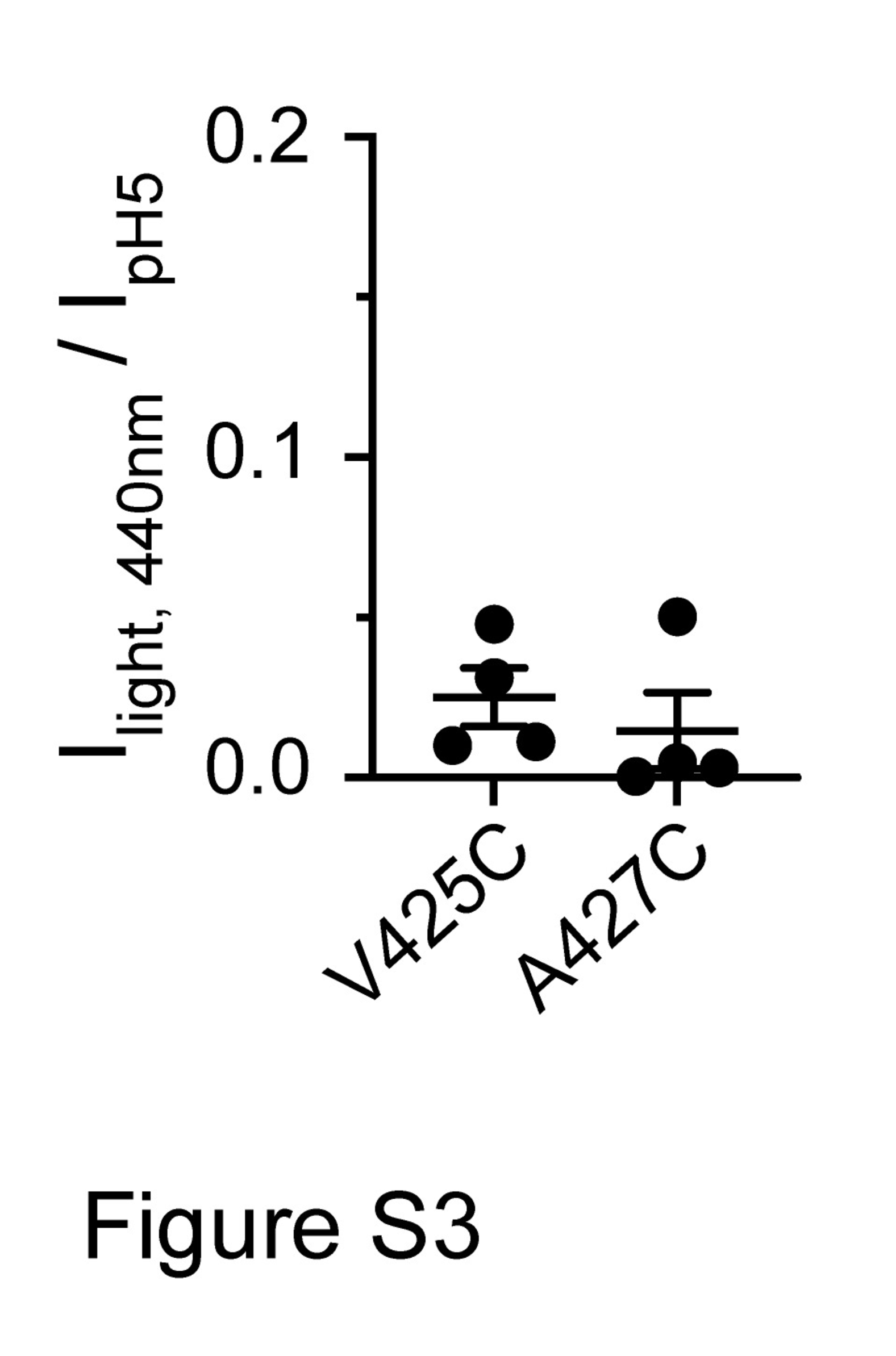

Supplement: S3 Fig — Cells expressing mutants were labelled with 10μM BMA for 12 min prior to the measurement. Data were obtained from whole-cell patch-clamp recording of transfected CHO cells, voltage-clamped to -60mV. Channels were exposed to pH5 or to 440nm light for 5s and 360nm light for 0.1s. (TIF) [file pone.0270762.s003.tif]

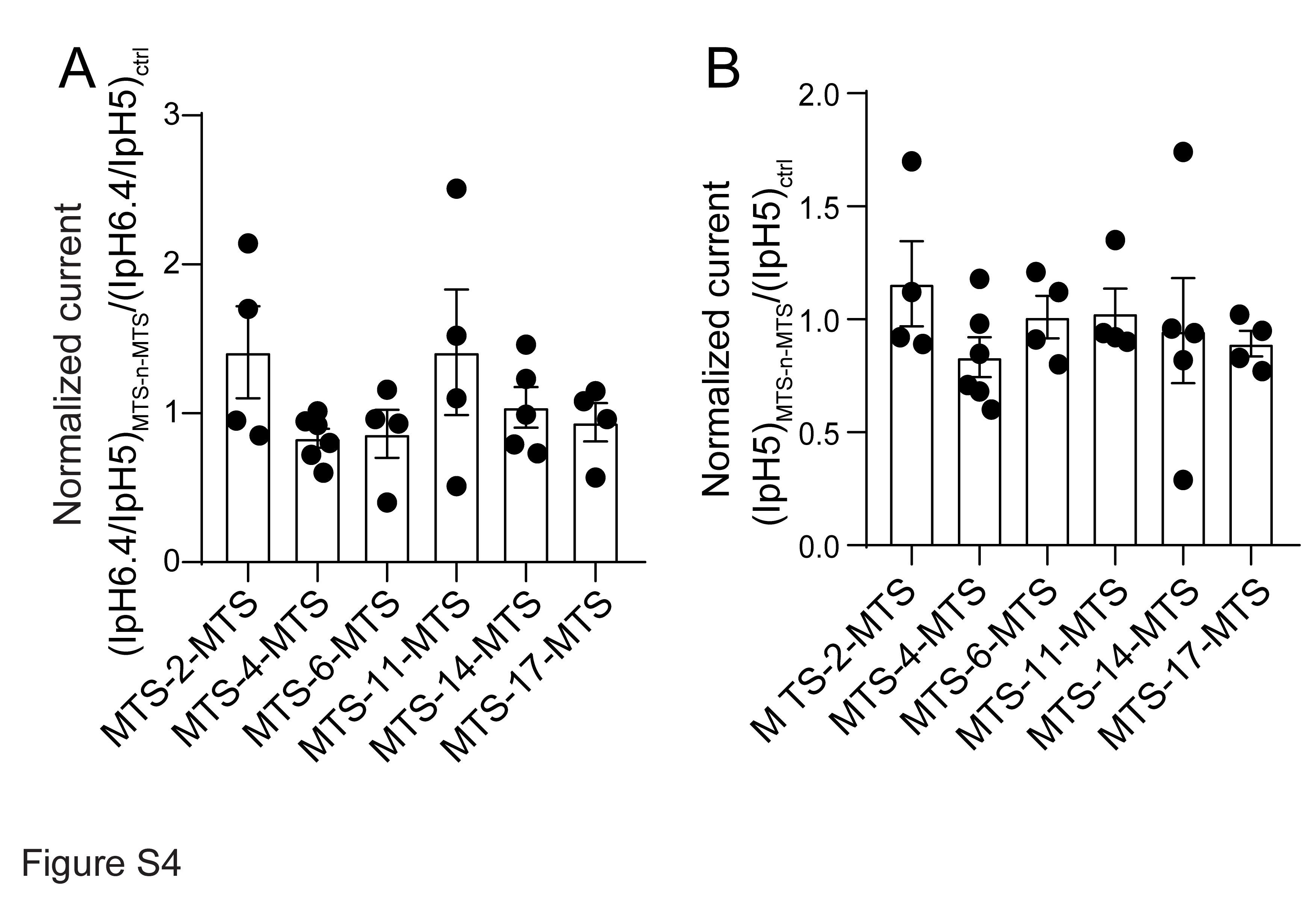

Supplement: S4 Fig — Data were obtained from two-electrode voltage-clamp of Xenopus oocytes expressing the WT ASIC1a clamped to -60mV. Currents were induced by exposure to pH6.4 and pH5.0 before and after exposure to MTS crosslinking reagents (1mM, 3min), in the same cell. The currents were normalized as indicated. (TIF) [file pone.0270762.s004.tif]

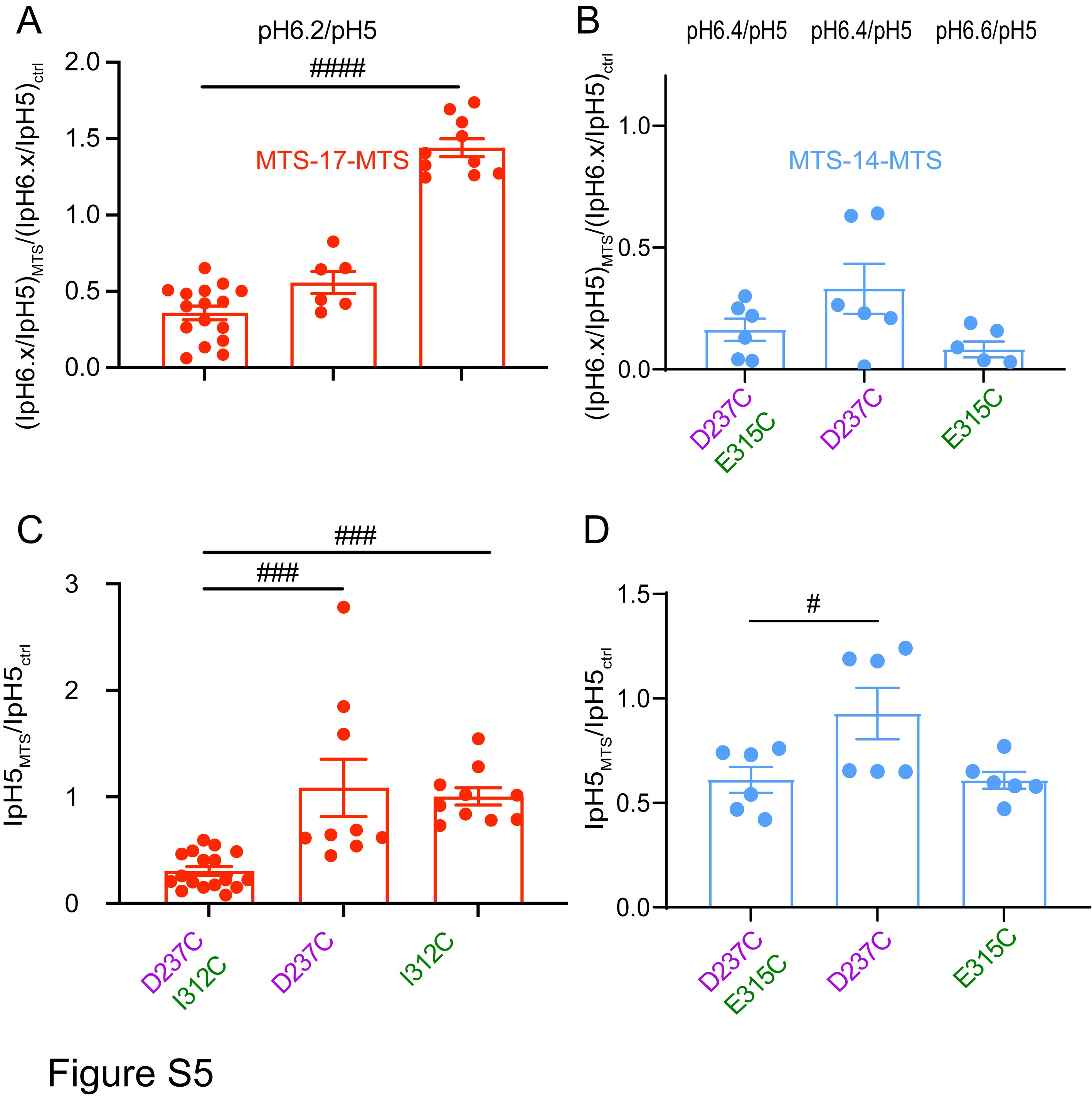

Supplement: S5 Fig — Data were obtained from two-electrode voltage-clamp of Xenopus oocytes expressing the indicated mutants, clamped to -60mV. Current ratios obtained from the indicated mutants after exposure to the MTS crosslinking reagent, normalized to the control measurement done before MTS reagent incubation. A-B, Ratio of (IpH6.x/IpH5)MTS/(IpH6.x/IpH5)ctrl and C-D, Ratio of IpH5MTS/IpH5ctrl. A, C, D237C/I312C, B, D, D237C/E315C. Statistical significance was determined by Ordinary one-way ANOVA and Dunnett’s post-hoc test, n = 6–17; #, p < 0.05, ###, p < 0.001, ####, p<0.0001. The pH conditions are the same as in Fig 10. The reagents were: With D237C/I312C and corresponding single mutants, MTS-17-MTS; With D237C/E315C and corresponding single mutants, MTS-14-MTS. (TIF) [file pone.0270762.s005.tif]
