# Supplementary material for: Probing conformational changes during activation of ASIC1a by an optical tweezer and by methanethiosulfonate-based cross-linkers
Source: PLoS One. 2022 Jul 8;17(7):e0270762. doi: 10.1371/journal.pone.0270762 (PMC9269482; doi:10.1371/journal.pone.0270762)
Supplement: S1 Table — The length of each MTS cross-linker or monovalent reagent was determined as the distance between the Sulphur atoms on both ends after the release of sulfinic acid (SO2CH3; for cross-linkers), and between the Sulphur atom and the other end of the molecule (monovalent MTS reagents). (PDF) [file pone.0270762.s006.pdf]

S1 Table. Length of MTS cross-linkers and monovalent reagents

MTS cross-linker	Length (Å)	Monovalent MTS	Length (Å)
MTS-2-MTS	6.3	MTSES	5.1
MTS-4-MTS	9.2	MTSEA-Biotin	10.9
MTS-6-MTS	12.3	MTS-PEO$_3$-Biotin	24.7
MTS-8-MTS	14.6		
MTS-10-MTS	15.8		
MTS-11-MTS	17.5		
MTS-14-MTS	21.1		
MTS-17-MTS	23.0		

The length of each MTS cross-linker or monovalent reagent was determined as the distance between the Sulphur atoms on both ends after the release of sulfinic acid (SO_2CH_3; for cross-linkers), and between the Sulphur atom and the other end of the molecule (monovalent MTS reagents).
