# Supplementary material for: Probing conformational changes during activation of ASIC1a by an optical tweezer and by methanethiosulfonate-based cross-linkers
Source: PLoS One. 2022 Jul 8;17(7):e0270762. doi: 10.1371/journal.pone.0270762 (PMC9269482; doi:10.1371/journal.pone.0270762)
Supplement: S2 Table — The indicated MTS cross-linkers were chosen for each mutant, based on the match between the cross-linker length and the distance between engineered Cys in the open (4NTW), desensitized (4NYK) and closed state (5WKU) models of human ASIC1a. Monovalent MTS reagents of similar length were chosen as controls. #, the length of the reagent matches the Cys-Cys distance of the desensitized state. (PDF) [file pone.0270762.s007.pdf]

S2 Table. Selection of MTS cross-linkers and monovalent MTS reagents for the different mutants

Mutant	Open state<br>MTS Cross-linker	Open state<br>MTS Monovalent	Closed state<br>MTS Cross-linker	Closed state<br>MTS Monovalent
R64C	MTS-14-MTS	MTS-PEO$_3$-Biotin	MTS-10-MTS[#]	MTSEA-Biotin
Y67C	MTS-17-MTS	-	MTS-10-MTS[#]	MTSEA-Biotin
H72C	MTS-14-MTS,<br>MTS-17-MTS	MTS-PEO$_3$-Biotin	MTS-10-MTS[#]	-
T419C	-	-	MTS-10-MTS[#]<br>MTS-14-MTS	-
E421C	MTS-14-MTS	-	MTS-10-MTS[#]	-
K423C	MTS-8-MTS<br>MTS-10-MTS	-	MTS-6-MTS[#]	MTSEA-Biotin
A425C	MTS-10-MTS	MTSEA-Biotin	MTS-4-MTS[#]<br>MTS-6-MTS[#]	MTSES
E427C	MTS-17-MTS	-	MTS-8-MTS<br>MTS-10-MTS[#]	
I428C	-	-	MTS-14-MTS[#]<br>MTS-17-MTS	MTS-PEO$_3$-Biotin
G430C	MTS-17-MTS	MTS-PEO$_3$-Biotin	MTS-4-MTS[#]<br>MTS-6-MTS<br>MTS-10-MTS	MTSEA-Biotin
G433C	MTS-10-MTS	-	MTS-2-MTS[#]<br>MTS-4-MTS	-
E97/D347	MTS-8-MTS[#]	MTSEA-Biotin	MTS-10-MTS	MTSEA-Biotin
E97/V354	MTS-11-MTS[#]	MTSEA-Biotin	MTS-14-MTS<br>MTS-17-MTS	MTS-PEO$_3$-Biotin
E97/E355	MTS-14-MTS[#]	MTS-PEO$_3$-Biotin	MTS-17-MTS	MTS-PEO$_3$-Biotin
E235/E355	MTS-10-MTS[#]	MTSEA-Biotin	MTS-17-MTS	MTS-PEO$_3$-Biotin
T236/D351	MTS-6-MTS[#]	MTSES	MTS-11-MTS	MTSEA-Biotin
D237/I312	MTS-11-MTS[#]	MTSEA-Biotin	MTS-17-MTS	MTS-PEO$_3$-Biotin
D237/E315	MTS-8-MTS[#]	MTSEA-Biotin	MTS-14-MTS	MTS-PEO$_3$-Biotin
D237/E355	MTS-6-MTS[#]	-	MTS-11-MTS<br>MTS-14-MTS	MTSEA-Biotin
K246/D347	MTS-11-MTS[#]	MTSEA-Biotin	MTS-14-MTS	MTS-PEO$_3$-Biotin
F257/D351	MTS-11-MTS[#]	MTSEA-Biotin	MTS-14-MTS	MTS-PEO$_3$-Biotin
D296/E359	MTS-8-MTS<br>MTS-10-MTS	MTSEA-Biotin	MTS-17-MTS[#]	MTS-PEO$_3$-Biotin

The indicated MTS cross-linkers were chosen for each mutant, based on the match between the cross-linker length and the distance between mutated residues (Cβ, and Cα for Gly) in the

open (4NTW [1]), desensitized (4NYK [1, 2]) and closed state (5WKU [3]) models of human ASIC1a. Monovalent MTS reagents of similar length were chosen as controls.

#, the length of the reagent matches the distance between mutated residues in the desensitized conformation.

References

1. Baconguis I, Bohlen CJ, Goehring A, Julius D, Gouaux E. X-ray structure of Acid-sensing ion channel 1-snake toxin complex reveals open state of a Na^{+}-selective channel. Cell. 2014;156(4):717-29.
2. Gonzales EB, Kawate T, Gouaux E. Pore architecture and ion sites in acid-sensing ion channels and P2X receptors. Nature. 2009;460(7255):599-604.
3. Yoder N, Yoshioka C, Gouaux E. Gating mechanisms of acid-sensing ion channels. Nature. 2018;555(7696):397-401.
