# Supplementary material for: Probing conformational changes during activation of ASIC1a by an optical tweezer and by methanethiosulfonate-based cross-linkers
Source: PLoS One. 2022 Jul 8;17(7):e0270762. doi: 10.1371/journal.pone.0270762 (PMC9269482; doi:10.1371/journal.pone.0270762)
Supplement: S3 Table — (PDF) [file pone.0270762.s008.pdf]

S3 Table. Summary of functional information obtained from previous studies using monovalent MTS reagents on ASIC1a mutants

MTS reagent	Mutation	Information
MTSET, MTSMT and MTSPT	mASIC1a-G428C	Direct activation of the channel at pH ≥ 7.0 [1]
MTSET	mASIC1a Y424C/G428C	
MTSET and MTSPTrEA	hASIC1a-G430C, hASIC2a-A427C	Direct activation of the channel at pH > 7.0 [2]
MTSET	hASIC1a-E315C, -D347C	Acidic shift in the pH dependence of activation and inhibition of peak amplitude [3]
MTSET	hASIC1a-E235C, -E355C	No shift in the pH dependence of activation [3]

References

1. Tolino LA, Okumura S, Kashlan OB, Carattino MD. Insights into the mechanism of pore opening of acid-sensing ion channel 1a. J Biol Chem. 2011;286(18):16297-307.
2. Gautschi I, van Bemmelen MX, Schild L. Proton and non-proton activation of ASIC channels. PloS one. 2017;12(4):e0175293.
3. Liechti LA, Berneche S, Bargeton B, Iwaszkiewicz J, Roy S, Michielin O, et al. A combined computational and functional approach identifies new residues involved in pH-dependent gating of ASIC1a. Journal of biological chemistry. 2010;285(21):16315-29.
