# Supplementary material for: Probing conformational changes during activation of ASIC1a by an optical tweezer and by methanethiosulfonate-based cross-linkers
Source: PLoS One. 2022 Jul 8;17(7):e0270762. doi: 10.1371/journal.pone.0270762 (PMC9269482; doi:10.1371/journal.pone.0270762)
Supplement: S1 Raw images — (PDF) [file pone.0270762.s009.pdf]

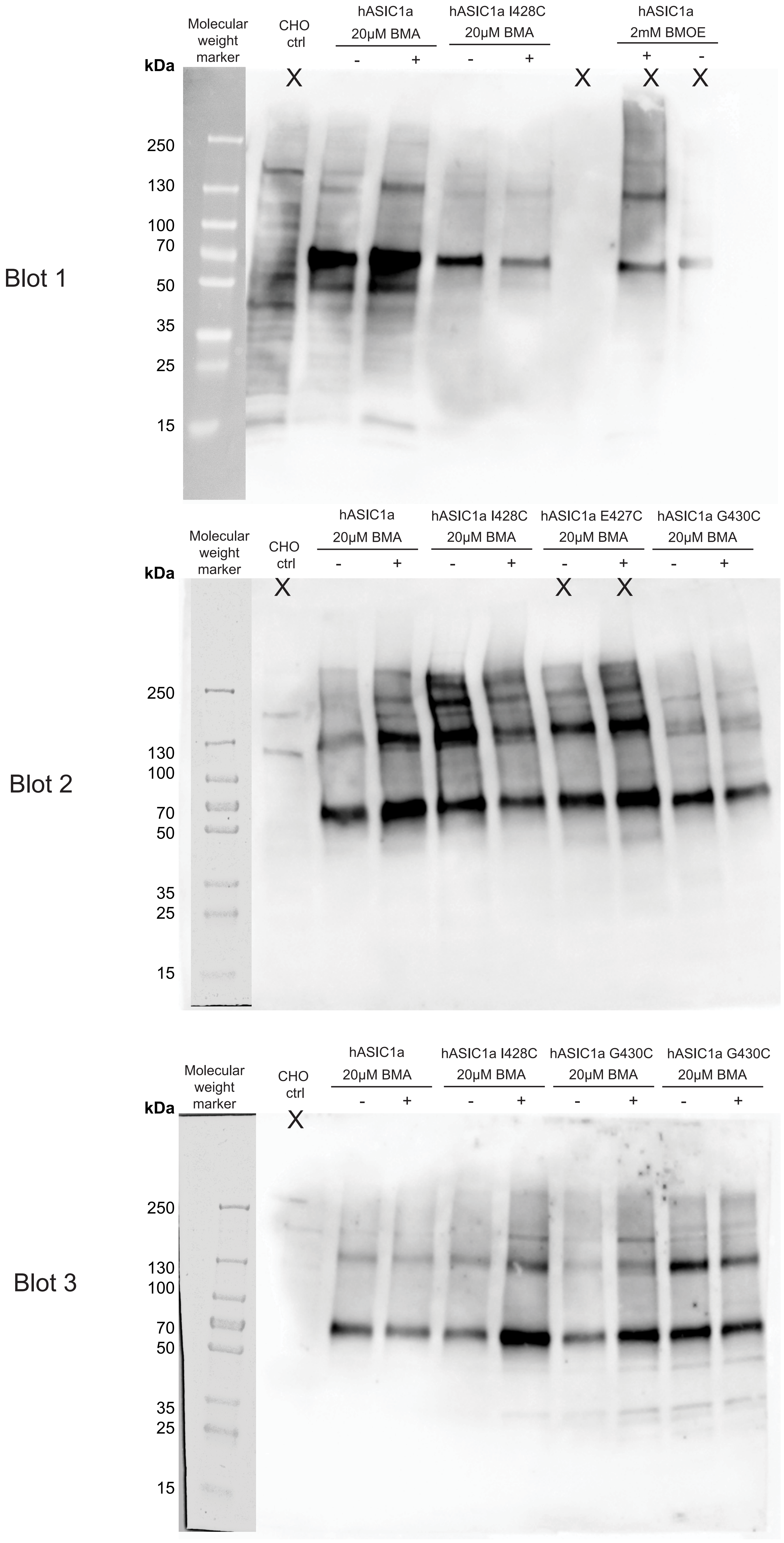

Figure 6A shows WT and I428C data from blot1 and G430C data from blot2.
“X” marks lanes not used for the analysis

S1_raw_images.pdf
